# Community-based prediction models of cardiovascular events, acute exacerbations and all-cause mortality in individuals with chronic obstructive pulmonary disease: a systematic review and meta-analysis on behalf of the International Cardiovascular and Respiratory Alliance

**DOI:** 10.1136/bmjresp-2025-003752

**Published:** 2026-02-27

**Authors:** Tobin Joseph, Keerthenan Raveendra, Mohammad Haris, Jasmin Kirupananthan, Amaan Aslam, Alexandra Mircescu, Ashmit Bhardwaj, Aidan Wong, Ramesh Nadarajah, David B Price, Mohit Bhutani, Chris Gale

**Affiliations:** 1 Leeds Institute of Cardiovascular and Metabolic Medicine, University of Leeds, Leeds, UK; 2 Leeds Institute for Data Analytics, University of Leeds, Leeds, UK; 3 Department of Cardiology, Leeds Teaching Hospitals NHS Trust, Leeds, UK; 4 Observational and Pragmatic Research Institute, Singapore; 5 University of Aberdeen Academic Primary Care Research Group, Aberdeen, UK; 6 Medicine, University of Alberta, Edmonton, Alberta, Canada

**Keywords:** COPD epidemiology, COPD Exacerbations

## Abstract

**Introduction:**

Preventable morbidity and mortality from chronic obstructive pulmonary disease (COPD) accrue from major adverse cardiovascular events (MACEs) and acute exacerbations of COPD (AECOPD). The study aims to summarise models for the prediction of these cardiopulmonary events in community-based settings.

**Methods:**

We searched for studies of multivariable models derived, validated or augmented for the prediction of cardiopulmonary events in COPD and used community-based data sources using MEDLINE and Embase from inception through 10 April 2025. Discrimination measures for the model with C-statistic data from ≥3 cohorts were pooled by Bayesian meta-analysis, and heterogeneity and risk of bias assessments were undertaken.

**Results:**

No models were identified that predicted cardiopulmonary events in COPD using community-based data. Of the 71 models included, 5 predicted cardiovascular events, 32 predicted AECOPD and 30 predicted all-cause mortality. None were eligible for meta-analysis for the prediction of cardiovascular events or AECOPD. For all-cause mortality, age, dyspnoea and airflow obstruction—surprise question (ADO-SQ) (0.763, 95% CI 0.533 to 0.942) and body mass index, airflow obstruction, dyspnoea score and exercise capacity (BODE) (0.753, 95% CI 0.583 to 0.907) demonstrated good prediction performance, while ADO (0.638, 95% CI 0.443 to 0.827) demonstrated adequate prediction performance. The risk of bias was high for 57.9% of studies, and none had clinical utility evaluated.

**Conclusions:**

Despite the high burden of MACE and AECOPD, there is an absence of community-based models that predict this composite outcome. Models to identify individuals with COPD at high risk of cardiopulmonary events could enable targeted clinical intervention.

**PROSPERO registration number:**

CRD420251026275.

**WHAT IS ALREADY KNOWN ON THIS TOPIC**

- Chronic obstructive pulmonary disease (COPD) is associated with a high burden of multimorbidity, particularly cardiovascular disease. Cardiopulmonary risk has recently been identified and defined by the International Cardiovascular and Respiratory Alliance, and it is unclear whether there are high-quality prediction models that predict cardiopulmonary risk in individuals with COPD.

**WHAT THIS STUDY ADDS**

- There are no prediction models that predict cardiopulmonary risk in patients with COPD. When split into cardiovascular disease, exacerbations and mortality, there are still few models that are limited by small sizes and little to no external validation outside of model development.

**HOW THIS STUDY MIGHT AFFECT RESEARCH, PRACTICE OR POLICY**

- This study highlights the need for the development of an accurate prediction model for cardiopulmonary risk, particularly one that is rigorously externally validated and tested prospectively.

## Introduction

 Chronic obstructive pulmonary disease (COPD) affects an estimated 480 million people globally, is the fourth leading cause of death worldwide and is projected to rapidly increase in prevalence.^1 2^ International guidelines recommend interventions that delay adverse outcomes, either as a direct consequence of COPD or as part of wider comorbidity.^1^ Preventable morbidity and mortality from COPD accrue not only from acute exacerbations of COPD (AECOPD) and worsening respiratory function but also from major adverse cardiovascular events (MACEs) in most patients with COPD.^3^ Accordingly, individuals with COPD are at increased cardiopulmonary risk.

The International Cardiovascular and Respiratory Alliance (ICRA) has defined cardiopulmonary events and cardiopulmonary risk, detailed an approach to the identification and management of cardiopulmonary risk and described current knowledge and evidence gaps in cardiopulmonary risk in COPD.^3^,^5^ One identified gap is the need for a prediction model for cardiopulmonary events in COPD. Such a tool could be used to identify individuals with COPD who may benefit from targeted interventions to reduce future cardiopulmonary events.

Therefore, we performed a systematic review and meta-analysis of prediction models of cardiopulmonary events in COPD that are applicable and have been validated in community-based cohorts. We aimed to synthesise the discriminatory ability of the identified prediction models and to determine whether any may be suitable for adoption into clinical practice.

## Methods

This systematic review has been reported in accordance with the Preferred Reporting Items for Systematic Reviews and Meta-Analyses (PRISMA) guidelines.^4^ It was registered on PROSPERO (CRD420251026275) and has been informed by the PRISMA statement and CHecklist for critical Appraisal and data extraction for systematic Reviews of prediction Modelling Studies (CHARMS).^4 5^

### Search strategy and inclusion criteria

We searched MEDLINE and Embase databases through the Ovid platform from inception to 10 April 2025 (online supplemental tables 1 and 2). A combination of keywords and subject headings related to cardiopulmonary events in COPD and prediction models was used, with records further limited to human studies. We conducted forward and backward citation searching for the included studies and previous systematic reviews. We removed duplicates using both EndNote’s duplicate identification strategy and manual removal.

To be eligible for inclusion, a study had to be an original study in human adults (≥18 years of age) with COPD and develop and/or validate a prediction model(s) for incident cardiopulmonary events based on multivariable analysis. Cardiopulmonary risk is defined as the risk of experiencing a cardiopulmonary event. Cardiopulmonary events are a composite term entailing moderate or severe COPD exacerbation, myocardial infarction, stroke, heart failure event, arrhythmias (atrial flutter, atrial fibrillation, ventricular tachycardia and ventricular fibrillation) or death due to any of these events.^6^ Systematic searching found no published models for the prediction of cardiopulmonary events. Thus, we extracted and reported on the components of cardiopulmonary risk—MACE, AECOPD and all-cause mortality. Articles were excluded if they only reported measures of association between risk factors and incident cardiopulmonary events rather than a complete prediction model, studied only a subset of the COPD population (eg, inpatients admitted with ongoing exacerbation) or incorporated variables not routinely available in community-based care (eg, galectin and Midregional pro-atrial natriuretic peptide (MR-pro-ANP)).

We uploaded records to a systematic review web application (Rayyan, Qatar Computing Research Institute). Four investigators (AB, AW, AM and MH) independently screened them for inclusion by title, abstract and full text. Disagreements were resolved by consultation with a further investigator (KR or TJ).

### Data extraction and quality assessment

Two investigators (AA and JK) independently extracted the data from the studies included. Data were drawn from the primary reference, unless stated otherwise. They then assessed each model in each study for risk of bias and applicability to the review question using the Prediction model Risk Of Bias Assessment Tool (PROBAST).^7^ Discrepancies between reviewers were resolved through additional reviews during group discussions with a third investigator (KR). The assessment of the certainty of evidence was conducted using the Grading of Recommendations Assessment, Development and Evaluation (GRADE) guidelines.^8^

To allow quantitative synthesis and assessment of the predictive performance of the models, we extracted measures of discrimination and calibration. To assess discrimination, we extracted data on the C-statistic or the area under the receiver operating characteristic, along with the corresponding 95% CI. If no CI was reported, we computed these using methods outlined by Debray *et al*.^9^ Calibration evaluates the accuracy of the model’s predicted probabilities, and we extracted all performance measures reported. We did not extract these metrics when reporting for artificial/synthetic data. We checked for the reporting of clinical utility of a model, namely, net benefit in the form of decision curve analysis or decision analytical modelling, which can be used to integrate the benefits and harms of using a model for clinical decision support. We also extracted cohort information, outcome definitions and the prediction variables used.

### Data synthesis and statistical analysis

We reported continuous variables as means±SD and categorical variables as percentages. Statistical significance was evaluated at 0.05 in all analyses. When a study reported performance in multiple cohorts and presented separate data for each cohort, we assessed model performance separately for each cohort within that study.

We performed a Bayesian meta-analysis of discrimination through a summary measure of the C-statistic and corresponding 95% CIs. This methodology was adopted, as frequentist methods are likely to encounter estimation problems in meta-analyses of prediction model performance due to validations and data being sparse. Additionally, frequentist methods can occasionally produce unreliable prediction intervals (PIs). Furthermore, Bayesian methods are useful when using sparse data to derive PIs.^9^ We calculated a 95% PI to depict the extent of between-study heterogeneity and to indicate a possible range for prediction model performance in a new validation.^9^ We categorised summary C-statistics of <0.60, 0.60–0.70, 0.70–0.80 and >0.80 a priori as inadequate, adequate, good and excellent, as defined by prior publications.^10^

The primary analysis assessed overall discrimination for models that had ≥3 validation cohorts with C-statistic data. In the subgroup analysis, a meta-analysis was performed for each model with ≥3 cohorts reporting C-statistic data while applying a uniform prediction window (eg, 1, 5 or 10 years), an important factor in translating risk models to clinical settings. We performed a sensitivity analysis where we excluded studies deemed high overall risk of bias by the PROBAST assessment. Statistical analyses were conducted in R using the metafor and metamisc packages (R Foundation for Statistical Computing, V.3.6.3).

### Patient and public involvement

Patients and public were not involved in the design, conduct or reporting of this study.

## Results

We identified 12 130 unique records, reviewed 49 full-text reports and included 38 studies (online supplemental figure 1 and tables 1 and 2).

### Characteristics of included studies

Five studies investigated prediction models for cardiovascular risk (incident atrial fibrillation, ischaemic heart disease and heart failure),^11^,^15^ comprising 12 cohorts. 20 studies investigated prediction models for AECOPD,^16^,^34^ comprising 27 cohorts. 13 studies investigated prediction models for all-cause mortality in COPD,^35^,^47^ comprising 19 cohorts. The number of included participants ranged from 286 to 1561 in studies for cardiovascular disease modelling, from 64 to 48 789 in studies for AECOPD and 61 to 54 990 in studies investigating prediction of all-cause mortality (table 1).

**Table 1:** Study characteristics of included EV studies

Author	Cohort	Types of cohort included	Participant number (n)	Outcome
Lovelace *et al*^41^	ECLIPSE	D/EV	2312	Mortality
Strand *et al*^36^	COPDGene; GOLD 0–4 and PRISm	D/EV	9074	Mortality
Strand *et al*^36^	SPIROMICS; GOLD 1–4	D/EV	NA	Mortality
Owusuaa *et al*^40^	Netherlands	D/IV/EV	358	Mortality
Bloom *et al*^42^	UK	D/IV/EV	54 990	Mortality
Horne *et al*^45^	SUMMIT	IV/EV	8304	Mortality
Horne *et al*^45^	Intermountain Cardiovascular	EV	9251	Mortality

COPD, chronic obstructive pulmonary disease; D, development; ECLIPSE, evaluation of copd longitudinally to identify predictive surrogate end-points; EV, external validation; GOLD, global initiative for chronic obstructive lung disease; IV, internal validation; PRISm, preserved ratio impaired spirometry; SPIROMICS, SubPopulations and InteRmediate Outcome Measures in COPD Study; SUMMIT, Study to Understand Mortality and Morbidity in COPD.

### Characteristics of included prediction models

**Figure 1:**
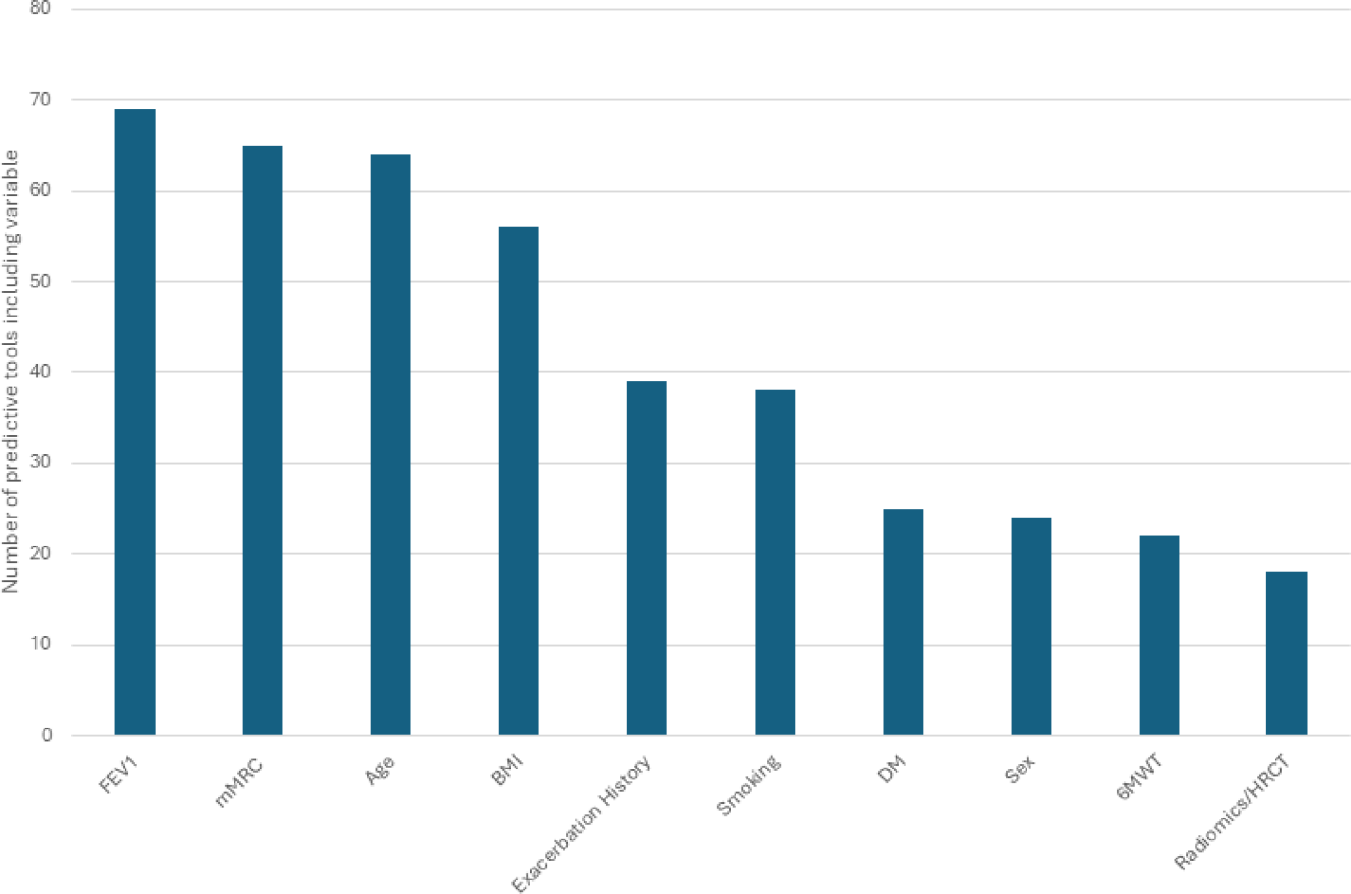
Overview of the 10 most used predictors for prediction models across cardiovascular risk, COPD exacerbations and all-cause mortality. BMI, body mass index; COPD, chronic obstructive pulmonary disease; DM, diabetes mellitus; FEV_1_, forced expiratory volume in 1 s; HRCT, high resolution CT; mMRC, modified Medical Research Council Dyspnoea Scale; 6MWT, 6 min walk test.

There were 71 unique multivariable models investigated across the three domains, including 9 for cardiovascular events, 32 for AECOPD and 30 for all-cause mortality (online supplemental tables 3–8). The 10 most commonly used variables for prediction are demonstrated in figure 1, and the full list of included variables is summarised in online supplemental tables 3–9. The most commonly used predictor variables for each domain are in online supplemental figures 2–4. The most frequently used variable was forced expiratory volume in 1 s (n=69), followed by the modified Medical Research Council Dyspnoea Score (n=65) and age (n=64). The most commonly used predictor variables for cardiovascular disease, AECOPD and all-cause mortality are presented in online supplemental figures 2–4, respectively.

### Risk prediction model performance among included cohorts

All studies reported a measure of discrimination ranging from 0.570 (95% CI 0.410 to 0.730)^11^ to 0.948 (95% CI 0.934 to 0.958)^15^ for prediction models of cardiovascular disease, 0.58 (95% CI 0.44 to 0.70)^26^ to 0.95 (95% CI 0.917 to 0.983)^29^ for models of AECOPD and from 0.55 (95% CI 0.31 to 0.78)^40^ to 0.915 (95% CI 0.886 to 0.944)^37^ for models predicting all-cause mortality (online supplemental tables 10–12). Three prediction models for cardiovascular disease were externally validated, but all were from the same study. 7 prediction models for AECOPD and 16 prediction models for all-cause mortality were also externally validated. The most frequently validated model for AECOPD was Dyspnoea, Obstruction, Smoking, Exacerbation; however, associations with AECOPD were more often reported than discrimination or calibration performance. The most frequently validated model for all-cause mortality was Age, Dyspnoea, Obstruction (ADO).

### Clinical utility and clinical impact of included risk prediction models

No studies included a clinical utility analysis, and no model was prospectively validated after forward citation searching.

### Risk of bias assessment

Overall, 22/38 (57.9%) studies were judged to be at high risk of bias (online supplemental tables 13–15 and figure 5). 80% of the cardiovascular risk prediction models, 60% of the AECOPD prediction models and 46% of the all-cause mortality prediction models were judged to be at high risk of bias, predominantly due to the method of analysis. There were no concerns for applicability for any of the models assessed (online supplemental tables 13–15). Egger’s test was performed (p=0.121), suggesting against publication bias (online supplemental figure 6).

### Meta-analysis

**Figure 2:**
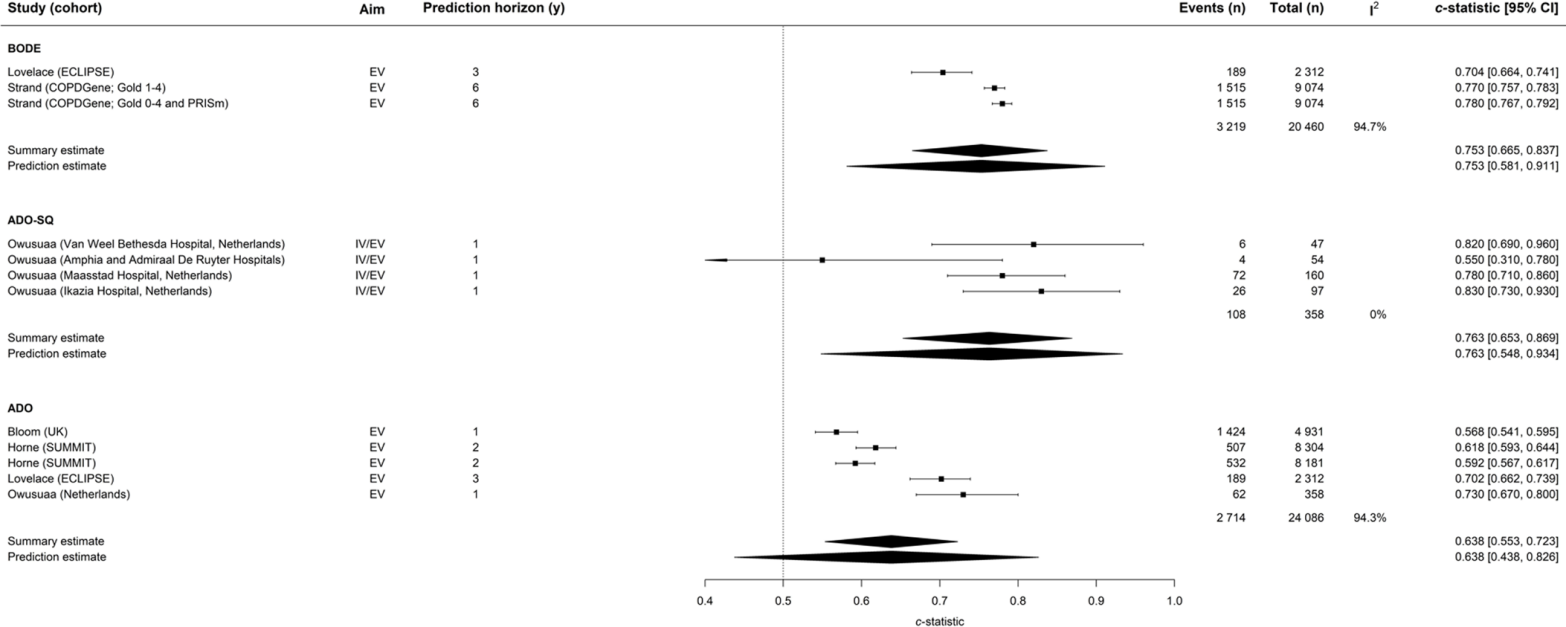
Forest plot of models developed for all-cause mortality restricted to results from EV studies only. ADO, age, dyspnoea and airflow obstruction; ADO-SQ, ADO—surprise question; BODE, body mass index, airflow obstruction, dyspnoea score and exercise capacity; BODEx, BODE and exacerbations; COPD, chronic obstructive pulmonary disease; ECLIPSE, evaluation of copd longitudinally to identify predictive surrogate end-points; EV, external validation; GOLD, global initiatilve for chronic obstructive lung disease; IV, internal validation; PRISm, preserved ratio impaired spirometry; SPIROMICS, SubPopulations and InteRmediate Outcome Measures in COPD Study; SUMMIT, Study to Understand Mortality and Morbidity in COPD;y, years.

**Figure 3:**
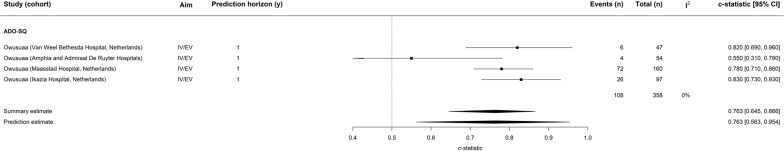
Forest plot of models developed for 1-year mortality and EV results only. ADO-SQ, age, dyspnoea and airflow obstruction—surprise question; EV, external validation; IV, internal validation; y, years.

For all-cause mortality, ADO-surprise question (ADO-SQ) (0.763, 95% CI 0.533 to 0.942) and body mass index, airflow obstruction, dyspnoea score and exercise capacity (BODE) (0.753, 95% CI 0.583 to 0.907) demonstrated good prediction performance while ADO (0.638, 95% CI 0.443 to 0.827) demonstrated adequate prediction performance (figure 2) when meta-analysing external validation (EV) cohorts. Both the BODE and ADO models demonstrated substantial heterogeneity (I^2^=94.7% and 94.3%, respectively), whereas the ADO-SQ demonstrated none (I^2^=0%). When restricted to a uniform prediction window and EV cohorts only, only the ADO-SQ model for 1-year prediction was eligible and demonstrated good prediction performance (0.762, 95% CI 0.549 to 0.939) (figure 3). This model demonstrated no heterogeneity (I^2^=0%). For COPD exacerbations or cardiovascular risk, no model was externally validated with reporting of prediction performance on enough occasions to enable meta-analysis.

### Sensitivity analyses

When excluding high-risk-of-bias studies for all-cause mortality, and restricting the analysis to EV results only, BODE demonstrated good prediction performance (0.753, 95% CI 0.570 to 0.902) and ADO demonstrated adequate prediction performance (0.663, 95% CI 0.386 to 0.911). Both analyses had substantial heterogeneity (I^2^=94.7% and 94.4%, respectively) (online supplemental figure 7). It was not possible to perform a meta-analysis for EV studies, nor to remove high-risk-of-bias studies and restrict the analysis to a uniform prediction horizon of 1 year.

When excluding high-risk-of-bias studies for all-cause mortality, BODE (0.753, 95% CI 0.588 to 0.910) demonstrated good prediction performance, ADO (0.665, 95% CI 0.451 to 0.866) demonstrated adequate prediction performance and BODE and exacerbations (0.535, 95% CI 0.177 to 0.900) demonstrated inadequate prediction performance (online supplemental figure 8). All models demonstrated substantial heterogeneity (I^2^=94.7%, 94.7% and 98.4%, respectively).

### Certainty of evidence

We assessed certainty of evidence using the GRADE tool.^48^ The initial certainty level of the included studies was ‘high’ because the relationship between predictors and outcomes was considered without any evidence of causality. However, this was downgraded to ‘low’ because of inconsistent results (high heterogeneity) and a high proportion of studies with a high risk of bias, despite limited concerns for applicability. Therefore, the final overall certainty is ‘low’.

## Discussion

To our knowledge, this is the first systematic review to investigate the utility of community-based prediction models to identify cardiopulmonary events in COPD. We identified no community-based models designed to predict incident cardiopulmonary events in COPD. When split into domains of cardiovascular disease, AECOPD and all-cause mortality, we found 71 models. Both the BODE and ADO-SQ models demonstrated good prediction performance for all-cause mortality, but meta-analysis of model performance for both the cardiovascular disease and AECOPD domains was not possible due to the absence of reported results.

### Comparison to previous studies

Previous studies have assessed prediction models for cardiovascular disease, COPD-related mortality and all-cause mortality separately, though none have focused on the prediction of cardiopulmonary events in COPD.^49 50^ Guerra *et al* conducted a systematic review of AECOPD prediction models,^50^ and Bellou *et al* conducted a systematic review of prediction models for all-cause mortality^49^; each found similar methodological concerns for clinical utility and, in particular, limited EV. Our review extends beyond the previous literature by demonstrating that few models have been developed for cardiovascular disease in COPD despite evidence suggesting that individuals with mild-moderate COPD are more likely to die of cardiovascular disease.^51^

### Clinical impact

Cardiovascular disease and associated risk factors in patients with COPD are frequently under-recognised, and cardiovascular risk and disease management are suboptimal.^52^ Notably, conventional models for the primary prevention of cardiovascular disease underestimate cardiovascular risk in COPD.^53^ Although QRISK4 has been developed in the UK to include COPD as an additional risk factor,^54^ there remains a need for a specific tool for the prediction of cardiopulmonary events in individuals with COPD. Prediction of the composite outcome of cardiopulmonary events in COPD could enable targeted diagnostics and preventative strategies in this high-risk population.^53^ This is in line with one of the identified evidence gaps by ICRA.^3^

None of the available models appears to have the requisite evidence base to be recommended for assessing cardiopulmonary risk in patients with COPD. Both BODE and ADO-SQ demonstrated good prediction performance, yet this was only in the all-cause mortality domain. BODE also had adequate to good prediction performance for AECOPD risk in two studies. However, BODE has not been tested for the prediction of cardiovascular events, nor has its clinical utility been prospectively evaluated. The ADO-SQ model was only validated in one country (the Netherlands), and the number of events in the studies was small, which might lead to an overestimated performance.

However, despite this, BODE could be considered most applicable, as it demonstrated adequate to good prediction performance for exacerbations and good prediction performance for all-cause mortality. Additionally, its simplicity could translate to higher implementation in clinical practice. It is possible that simpler, parsimonious models can have higher clinical utility, as more complex models can be limited by data missingness. Simpler prediction models could be evaluated in future research.

Lack of EV and prospective evaluation are common shortcomings in prediction modelling research.^55^ Across the three domains of cardiopulmonary risk, summarised prediction models shared common predictor variables. This suggests that morbidity in COPD is increasing and that these three types of adverse events all likely share common risk factors (such as age, smoking status and prior cardiovascular disease) and pathobiology. This means that it should be possible to develop a prediction model for cardiopulmonary events in COPD, but such a model will require EV and prospective testing to demonstrate utility. Additionally, our assessment of the certainty of evidence was ‘low’, suggesting that further studies in this domain were likely to significantly alter the effect estimates in each domain, and the creation of a cardiopulmonary event prediction model would affect the overall outcome.

### Limitations

There are several limitations to our work. First, the populations included in the analyses are derived and validated in very heterogeneous populations, with a wide variation in the number of participants used to create and validate the models and the number of events used to train the model. We included both prospective and retrospective studies, which might introduce bias in the diagnosis of certain events, as well as selection bias, to help train models. Additionally, we did not perform meta-regression or subgroup meta-analysis because of the lack of individual patient data, and thus, this analysis could be prone to ecological bias.^56^ We also could not analyse model calibration performance due to under-reporting. Furthermore, the meta-analyses frequently demonstrated substantial heterogeneity, which increases the likelihood that new prediction models would significantly alter our results.

## Conclusions

Presently, there are no evidence-based models for the prediction of cardiopulmonary risk in COPD. Given the significant impacts of cardiopulmonary events on COPD outcomes, there is an urgent need for further research to develop, externally validate and prospectively test a prediction model specifically for cardiopulmonary risk in patients with COPD.

## Supplementary material

10.1136/bmjresp-2025-003752 online supplemental file 1

## Data Availability

Data are available upon reasonable request.
