# Supplementary material for: Community-based prediction models of cardiovascular events, acute exacerbations and all-cause mortality in individuals with chronic obstructive pulmonary disease: a systematic review and meta-analysis on behalf of the International Cardiovascular and Respiratory Alliance
Source: BMJ Open Respir Res. 2026 Feb 27;13(1):e003752. doi: 10.1136/bmjresp-2025-003752 (PMC12958919; doi:10.1136/bmjresp-2025-003752)
Supplement: online supplemental file 1 [file bmjresp-13-1-s001.pdf]

Community-based prediction models of cardiovascular events, acute exacerbations and all-cause mortality in individuals with chronic obstructive pulmonary disease: systematic review and meta-analysis on behalf of the International Cardiovascular and Respiratory Alliance

Supplementary Material

T Joseph*, K Raveendra*, M Haris, J Kirupananthan, A Aslam, A Mircescu, A Bhardwaj, A Wong, R Nadarajah, DB Price, M Bhutani, CP Gale

Contents

SUPPLEMENTARY METHODS 3
- Formulation of research question using CHARMS (CHecklist for critical Appraisal and data extraction for systematic Reviews of prediction Modelling Studies)[1] 3
- Bayesian meta-analysis of *c*-statistic / AUROC 4
- Supplementary Table 1 Search Strategy Results for Medline 5
- Supplementary Table 2 Search Strategy Results for Embase and Embase Classic 7

SUPPLEMENTARY RESULTS 9
- Supplementary Table 3: Cohort description and outcomes with definitions 9
- Supplementary Table 4: Demographic and environmental variables used in the models 20
- Supplementary Table 5: Respiratory variables used in the models 24
- Supplementary Table 6: Cardiovascular variables used in the models 27
- Supplementary Table 7: Biochemical and imaging variables used in the models 29
- Supplementary Table 8: Miscellaneous variables used in prediction models 31
- Supplementary Table 9: Indices used in prediction models 32
- Supplementary Table 10: Performance metrics of prediction models for cardiovascular events 34
- Supplementary Table 11: Performance metrics of prediction models for exacerbations 35
- Supplementary Table 12: Performance metrics of prediction models for all-cause mortality 37
- Supplementary Table 13: Risk of Bias and Applicability Assessment for Each PROBAST Domain for studies predicting cardiovascular events 39
- Supplementary Table 14: Risk of Bias and Applicability Assessment for Each PROBAST Domain for studies predicting exacerbations 40
- Supplementary Table 15: Risk of Bias and Applicability Assessment for Each PROBAST Domain for studies predicting all-cause mortality 41
- Supplementary Figure 1: PRISMA Flow diagram demonstrating study selection 42
- Supplementary Figure 2: Overview of predictor variables used more than once models investigating cardiovascular outcomes 43

Supplementary Figure 3: Overview of predictor variables used more than once models investigating exacerbations 44

Supplementary Figure 4: Overview of predictor variables used more than once models investigating mortality 44

Supplementary Figure 5: Risk of Bias assessment for prediction models 46

Supplementary Figure 6: Funnel Plot assessing publication bias 47

Supplementary Figure 7: Sensitivity analysis for external validation results only for all-cause mortality, excluding studies at high risk of bias. 48

Supplementary Figure 8: Sensitivity analysis for all-cause mortality restricted to studies with low or unclear risk of bias for participants domain of PROBAST 49

SUPPLEMENTARY METHODS

Formulation of research question using CHARMS (CHecklist for critical Appraisal and data extraction for systematic Reviews of prediction Modelling Studies)[1]

CHARMS key items to guide framing of review, search strategy and study inclusion and exclusion criteria	Comments for this systematic review
Prognostic versus diagnostic prediction model	Prediction model
Intended scope of the review	Models to identify adverse cardiopulmonary events in stable COPD patients
Types of Prediction modelling studies	Prediction model development without external validation in independent data, prediction model development with external validation in independent data, external model validation, possibly with model augmentation
Target population to whom the prediction model applies	Adults who have experienced a cardiopulmonary event[2]
Outcome to be predicted	Any cardiopulmonary event[2]
Time span of prediction	Any time interval
Intended moment of using the model	Models to be used in adults who have experienced stroke to predict the risk of development/or identification of underlying atrial fibrillation in the future, and to inform targeted screening and/or secondary prevention

Bayesian meta-analysis of *c*-statistic / AUROC

All Bayesian meta-analysis models assume random effects by default. Results are based on the posterior median. Prediction intervals are directly obtained from the corresponding posterior quartiles. The standard model for random effects meta-analysis assumes that the 'true' performance is normally distributed within and across studies.[3] Within-study normality of performance estimates can be justified with this selection of included studies because they are all large. *Snell et al.* showed that the between-study distribution of the *c*-statistic on the original scale is not normally distributed when there is variability in the predictor effect across studies (which is likely in this selection of studies as they include different populations and adopt slightly different definitions for predictors).[3] They found that the logit scale is more appropriate for the estimation of prediction interval. Consequently, we used the "valmeta" function of the "metamisc" package in R software which applies a logit transformation to the *c*-statistic before the calculation of the summary *c*-statistic and prediction interval [4].

For appropriate prior distributions, we borrowed from earlier work by *Debray et al.* which recommended a half Student-*t* distribution with location m, scale σ, and v degrees of freedom where we set $m = 0$ and define σ equal to the largest empirical value of $\hat{\tau}$ (to allow for more extreme values of heterogeneity)[5]. These hyperparameter values allow us to penalise the extent of between-study heterogeneity when the number of included validation studies is low [5]. Further, we also used $v = 3$ to ensure that the variance $\sigma^2\, v/(v\text{-}2)$ exists and samples of τ were truncated above 10 to rule out unreasonable values. Thus the resulting priors are given as τdiscr ~ Student−$t(0, 0.5^2, 3)T[0.10]$ which has been shown to allow for large but realistic values for between-study heterogeneity.[5]

Supplementary Table 1 Search Strategy Results for Medline

#	Query	Results from April 10th, 2025
1	predict* model/ or statistical model/ or forecast model/ or predictive algorithm/ or prognosis model/ or prognostic model/ or risk predict* model/ or risk assessment model/ or predictive framework/ or forecasting model/ or prediction model/ or prediction algorithm/ or risk modeling/ or risk prediction/ or prognostic algorithm/ or prognostic framework/ or machine learning model/ or artificial intelligence model/ or deep learning model/ or decision support model/ or clinical prediction rule/ or predictive analytics/ or diagnostic model/ or outcome prediction model/ or computational model/ or statistical prediction/ or risk stratification model/ or health risk model/ or patient risk model/ or clinical forecast/ or clinical decision support/ or algorithmic model/ or predictive analytics/ or model validation/ or model calibration/ or risk scoring system/ or outcome forecasting/ or health prediction model/ or AI-driven model/ or statistical forecasting/ or data-driven model/ or predictive scoring/ or prediction rule/	176825
2	(predict* model or statistical model or forecast model or predictive algorithm or prognosis model or prognostic model or risk predict* model or risk assessment model or predictive framework or forecasting model or prediction model or prediction algorithm or risk modeling or risk prediction or prognostic algorithm or prognostic framework or machine learning model or artificial intelligence model or deep learning model or decision support model or clinical prediction rule or predictive analytics or diagnostic model or outcome prediction model or computational model or statistical prediction or risk stratification model or health risk model or patient risk model or clinical forecast or clinical decision support or algorithmic model or predictive analytics or model validation or model calibration or risk scoring system or outcome forecasting or health prediction model or AI-driven model or statistical forecasting or data-driven model or predictive scoring or prediction rule).ti,ab.	142756
3	1 or 2	305880
4	ROC curve/ or stratification/ or discrimination r c-statistic/ or c-statistic/ or area under the curve/ or calibration/ or indices/ or algorithm/ or multivariable.mp. or prognostic framework/ [mp=title, book title, abstract, original title, name of substance word, subject heading word, floating sub-heading word, keyword heading word, organism supplementary concept word, protocol supplementary concept word, rare disease supplementary concept word, unique identifier, synonyms, population supplementary concept word, anatomy supplementary concept word]	644463
5	(ROC curve or stratification or discrimination r c-statistic or c-statistic or area under the curve or calibration or indices or algorithm or multivariable or prognostic framework).ti,ab.	1030316
6	4 or 5	1294049
7	3 or 6	1472123
8	myocardial infarction*/ or MI/ or heart attack*/ or ACS/ or acute coronary syndrome/ or cardiac infarction/ or heart infarction/ or acute MI/ or acute myocardial infarction/ or STEMI/ or non-STEMI/ or NSETMI/ or ischemic heart disease/ or coronary event/ or heart ischemia/ or myocardial ischemia.mp. [mp=title, book title, abstract, original title, name of substance word, subject heading word, floating sub-heading word, keyword heading word, organism supplementary concept word, protocol supplementary concept word, rare disease supplementary concept word, unique identifier, synonyms, population supplementary concept word, anatomy supplementary concept word]	137986
9	(myocardial infarction* or MI or heart attack* or ACS or acute coronary syndrome or cardiac infarction or heart infarction or acute MI or acute myocardial infarction or STEMI or non-STEMI or NSETMI or coronary artery syndrome or cardiac ischemia or ischemic heart disease or coronary event or heart ischemia or myocardial ischemia).ti,ab.	349761
10	8 or 9	417937
11	AFIB/ or atrial fibrillation/ or AF/ or atrial flutter/ or ventricular fibrillation/ or VFIB/ or VT/ or ventricular tachycardia/	112365
12	(AFIB or atrial fibrillation or AF or atrial flutter or ventricular fibrillation or VFIB or VT or ventricular tachycardia).ti,ab.	175507
13	11 or 12	201712
14	stroke/ or cerebrovascular insult/ or CVI/ or cerebrovascular accident/ or CVA/ or brain attack/ or transient ischemic attack/ or TIA/ or mini-stroke/ or transient ischemia/ or transient cerebral ischemia/ or transient cerebral ischemic attack/ or TIA event/ or brief ischemic attack/ or brief cerebral ischemic attack/ or transient cerebrovascular event/ or transient stroke/ or TIA attack/ or temporary brain ischemia/ or transient	161856

	brain attack/ or transient ischemic stroke/ or transient cerebrovascular attack/ or transient cerebrovascular insult/	
15	(stroke or cerebrovascular insult or CVI or cerebrovascular accident or CVA or brain attack or transient ischemic attack or TIA or mini-stroke or transient ischemia or transient cerebral ischemia or transient cerebral ischemic attack or TIA event or brief ischemic attack or brief cerebral ischemic attack or transient cerebrovascular event or transient stroke or TIA attack or temporary brain ischemia or transient brain attack or transient ischemic stroke or transient cerebrovascular attack or transient cerebrovascular insult).ti,ab.	359525
16	14 or 15	388429
17	heart failure/ or HF/ or congestive heart failure/ or CHF/ or cardiac failure/ or congestive cardiac failure/ or CCF/	156177
18	(heart failure or HF or congestive heart failure or CHF or cardiac failure or congestive cardiac failure or CCF).ti,ab.	281532
19	17 or 18	320717
20	adverse cardiopulmonary event*/ or cardiopulmonary complication*/ or COPD exacerbation/ or respiratory event*/ or pulmonary complication*/	0
21	(adverse cardiopulmonary event* or cardiopulmonary complication* or COPD exacerbation or respiratory event* or pulmonary complication*).ti,ab.	16846
22	20 or 21	16846
23	incident/ or incidence/ or prevalence/ or epidemiology/ or occurrence/ or frequency/ or morbidity/ or commonness/ or distribution/	705833
24	(incident or incidence or prevalence or epidemiology or occurrence or frequency or morbidity or commonness or distribution).ti,ab.	4611802
25	23 or 24	4770200
26	death/ or mortality/ or morbidity/ or fatality/ or survival/	108307
27	(death or mortality or morbidity or fatality or survival).ti,ab.	3053710
28	26 or 27	3085729
29	10 or 13 or 16 or 19 or 22 or 25 or 28	7573541
30	COPD/ or chronic obstructive pulmonary disease/ or chronic bronchitis/ or emphysema/ or COAD/ or chronic obstructive airway disease/ or COLD/ or chronic obstructive lung disease/ or chronic lung disease/ or chronic airflow limitation/ or pulmonary disease/ or Chronic Obstructive/	193817
31	(COPD or chronic obstructive pulmonary disease or chronic bronchitis or emphysema or COAD or chronic obstructive airway disease or COLD or chronic obstructive lung disease or chronic lung disease or chronic airflow limitation or Pulmonary Disease, Chronic Obstructive).ti,ab.	285279
32	30 or 31	387660
33	(animal* or non-human* or nonhuman*).ti,ab.	1357957
34	animal model*.ti,ab.	226258
35	(rodent* or rat* or mice or mouse).ti,ab.	8715450
36	experiment* animal.ti,ab.	11914
37	33 or 34 or 35 or 36	9408939
38	7 and 29 and 32	8558
39	38 not 37	4096
40	limit 39 to dt=19640101-20250410 [January 1st, 1964 to April 10th, 2025]	3859

Supplementary Table 2 Search Strategy Results for Embase and Embase Classic

#	Query	Results from April 10th, 2025
1	prediction model/ or statistical model/ or prognostic model/ or risk prediction/ or machine learning model/ or artificial intelligence model/ or decision support model/ or clinical prediction rule/ or diagnostic model/ or outcome prediction model/ or risk stratification model/ or clinical decision support/ or model validation/ or model calibration/ or risk scoring system/ or health prediction model/ or AI-driven model/ or predictive scoring/ 182113	182113
2	(prediction model or statistical model or prognostic model or risk prediction or machine learning model or artificial intelligence model or decision support model or clinical prediction rule or diagnostic model or outcome prediction model or risk stratification model or clinical decision support or model validation or model calibration or risk scoring system or health prediction model or AI-driven model or predictive scoring).ti,ab,kw.	129730
3	1 or 2	302863
4	ROC Curve/ or Stratification/ or Discrimination/ or Calibration/ or indexes/ or Algorithms/ or Multivariate Analysis/ or Prognosis/	1680475
5	(ROC Curve or Stratification or Discrimination or Calibration or indexes or Algorithms or Multivariate Analysis or Prognosis).ti,ab,kw.	1989657
6	4 or 5	2795728
7	3 or 6	2994285
8	myocardial infarction/ or MI/ or heart attack/ or ACS/ or acute coronary syndrome/ or cardiac infarction/ or heart infarction/ or acute MI/ or acute myocardial infarction/ or STEMI/ or non-STEMI/ or NSTEMI/ or ischemic heart disease/ or ischaemic heart disease/ or coronary event/ or heart ischemia/ or heart ischaemia/ or myocardial ischemia/ or myocardial ischaemia/	754992
9	(myocardial infarction or MI or heart attack or ACS or acute coronary syndrome or cardiac infarction or heart infarction or acute MI or acute myocardial infarction or STEMI or non-STEMI or NSTEMI or ischemic heart disease or ischaemic heart disease or coronary event or heart ischemia or heart ischaemia or myocardial ischemia or myocardial ischaemia).ti,ab,kw.	583627
10	8 or 9	900983
11	AFIB/ or atrial fibrillation/ or AF/ or atrial flutter/ or ventricular fibrillation/ or VFIB/ or VT/ or ventricular tachycardia/ or VF/	320740
12	(AFIB or atrial fibrillation or AF or atrial flutter or ventricular fibrillation or VFIB or VT or ventricular tachycardia or VF).ti,ab,kw.	327250
13	11 or 12	427158
14	stroke/ or cerebrovascular insult/ or CVI/ or cerebrovascular accident/ or CVA/ or brain attack/ or transient ischemic attack/ or TIA/ or mini-stroke/ or transient ischemia/ or transient cerebral ischemia/ or transient cerebral ischemic attack/ or TIA event/ or brief ischemic attack/ or brief cerebral ischemic attack/ or transient cerebrovascular event/ or transient stroke/ or TIA attack/ or temporary brain ischemia/ or transient brain attack/ or transient ischemic stroke/ or transient cerebrovascular attack/ or transient cerebrovascular insult/	485147
15	(stroke or cerebrovascular insult or CVI or cerebrovascular accident or CVA or brain attack or transient ischemic attack or TIA or mini-stroke or transient ischemia or transient cerebral ischemia or transient cerebral ischemic attack or TIA event or brief ischemic attack or brief cerebral ischemic attack or transient cerebrovascular event or transient stroke or TIA attack or temporary brain ischemia or transient brain attack or transient ischemic stroke or transient cerebrovascular attack or transient cerebrovascular insult).ti,ab,kw.	590540
16	14 or 15	726543
17	heart failure/ or HF/ or congestive heart failure/ or CHF/ or cardiac failure/ or congestive cardiac failure/ or CCF/	463947
18	(heart failure or HF or congestive heart failure or CHF or cardiac failure or congestive cardiac failure or CCF).ti,ab,kw.	490012
19	17 or 18	639623
20	adverse cardiopulmonary event*/ or cardiopulmonary complication*/ or COPD exacerbation/ or respiratory event*/ or pulmonary complication*/	2
21	(adverse cardiopulmonary event* or cardiopulmonary complication* or COPD exacerbation or respiratory event* or pulmonary complication*).ti,ab,kw.	29308
22	20 or 21	29310
23	incident/ or incidence/ or prevalence/ or epidemiology/ or occurrence/ or frequency/	1971699

24	(incident or incidence or prevalence or epidemiology or occurrence or frequency).ti,ab,kw.	4780862
25	23 or 24	5318278
26	death/ or mortality/ or fatality/ or survival/	1664886
27	(death or mortality or fatality or survival).ti,ab,kw.	4316160
28	26 or 27	4704059
29	10 or 13 or 16 or 19 or 22 or 25 or 28	10318834
30	COPD/ or chronic obstructive pulmonary disease/ or chronic bronchitis/ or emphysema/ or COAD/ or chronic obstructive airway disease/ or COLD/ or chronic obstructive lung disease/ or chronic lung disease/ or chronic airflow limitation/ or pulmonary disease/ or Chronic Obstructive/	400445
31	(COPD or chronic obstructive pulmonary disease or chronic bronchitis or emphysema or COAD or chronic obstructive airway disease or COLD or chronic obstructive lung disease or chronic lung disease or chronic airflow limitation or Pulmonary Disease, Chronic Obstructive).ti,ab,kw.	437644
32	30 or 31	645734
33	(animal* or non-human* or nonhuman*).ti,ab,kw.	1818955
34	animal model*.ti,ab.	303620
35	(rodent* or rat* or mice or mouse).ti,ab,kw.	11783089
36	experiment* animal.ti,ab.	17194
37	(Case Report or Case Reports or (Case Series or Case Series) or Editorials or Letters).ti,ab. or (Abstract or Abstracts).ti,ab,kw.	1269845
38	33 or 34 or 35 or 36 or 37	13685727
39	7 and 29 and 32	33734
40	39 not 38	16377
41	limit 40 to dd=19460101-20250410 [January 1st, 1946 to April 10th, 2025]	15140

SUPPLEMENTARY RESULTS

Supplementary Table 3: Cohort description and outcomes with definitions

Author	Cohort	Cohort Description	Types of cohort included	Outcome	Outcome Definition
Krishnan et al[6]	CanCOLD	prospective longitudinal cohort study, tracking 1800 subjects with assessment at baseline, 18 months, 3 years and beyond following the same scheme	EV	CVD	NA
Lin et al[7]	Shanghai/Zhejiang	1661 patients with confirmed COPD by pulmonary function tests (PFT) at three centers between September 2015 and April 2023. Inclusion criteria were as follows: (1) COPD confirmed by PFT; (2) PFT and chest CT within 2 weeks; (3) complete thin-slice (1 mm) chest CT images.	D, IV, EV	CVD	10 definitions, as all diagnosis of hypertension, coronary artery disease (angina, (sub)acute myocardial infarction, acute or chronic ischemic heart diseases), and heart failure
Huang et al[8]	Second Affiliated Hospital of Guilin Medical College	retrospective study analyzed patients with COPD who were admitted to the Second Affiliated Hospital of Guilin Medical College (Guilin, Guangxi Zhuang Autonomous Region, P.R. China) between January 2020 and May 2022.	D/IV	CVD	presence of AF on surface electrocardiography or single-lead electrocardiographic recording lasting >30 sec

Qu et al[9]	NHANES	cross-sectional study collected the data of 1199 COPD patients in the NHANES database from 2007 to 2018.	D	CVD	patients with at least one myocardial infarction, congestive heart failure, angina pectoris, or a history of coronary heart disease
Shi et al[10]	NHANES	data were extracted from NHANES (2007–2012) database. A total of 3226 COPD adults aged 40 to 79 years were enrolled in this study	IV	CVD	determined respectively according to the questions "Ever told you had angina or heart failure", "Ever told you had heart attack", and "Has a doctor or other health professional ever told you that you had coronary heart disease".
Hussein et al[11]	Swiss COPD cohort	Swiss COPD cohort from 2014 and 2022.	D/IV	Exacerbation	estimated the overall sample mean cumulative function (MCF), which is the average number of cumulative

					exacerbations experienced by an individual in the study at each point in time,
Adibi et al[12]	ECLIPSE	patients with COPD, without previous or existing history of asthma, and who had at least one exacerbation over the past 12 months. Then externally validated the model in patients with COPD regardless of their exacerbation history and in a subset of patients with COPD with at least one exacerbation over the past 12 months.	D / EV	Exacerbation	acute episode of intensified symptoms that required additional therapy
Safari et al[13]	TORCH	patients with COPD, without previous or existing history of asthma, and who had at least one exacerbation over the past 12 months. Then externally validated the model in patients with COPD regardless of their exacerbation history and in a subset of patients with COPD with at least one exacerbation over the past 12 months.	EV	Exacerbation	acute episode of intensified symptoms that required additional therapy
Samp et al[14]	USA	retrospective health insurance claims data from January 1, 2004, through December 31, 2014, from the Truven Health MarketScan Commercial Claims and Encounters and Medicare Supplemental databases	D	Exacerbation	Inpatient exacerbations - hospitalization with a primary diagnosis code for COPD. Outpatient and ED exacerbations - outpatient or ED visits with a diagnosis code for COPD and prescription claims

					for an oral antibiotic or oral corticosteroid 5 days before or after the visit
Singla et al[15]	COPDGene	COPDGene includes 10 300 baseline participants, all of which were either current or former smokers. Each participant performed spirometry and had a high-resolution inspiratory and expiratory CT scan, using a standardized protocol	D / IV	Exacerbation	In last year, experienced at least one episode of increased dyspnea, cough or sputum production, resulting in admission or change of treatment plan.
Wu et al[16]	Taiwan	adult patients with COPD (20 years of age or older) who were not implanted with a pacemaker and who were not pregnant.	D / IV	Exacerbation	acute worsening of respiratory symptoms, resulting in additional therapy
Bertens et al[17]	Utrecht General Practice Network, Netherlands	patients from primary care enlisted with a general practitioner's (GP's) diagnosis of COPD and aged 65 years or over were selected from 51 general practices in the Netherlands between April 2001 and June 2003.	D / IV	Exacerbation	symptomatic deterioration requiring pulsed oral steroid use or hospitalization
Marin et al[18]	Tenerife/Zaragoza, Spain	subjects with a diagnosis of COPD were recruited between January 1997 and June 2002 at the outpatient pulmonary	EV	Exacerbation	sustained worsening of baseline respiratory

		clinics of two tertiary teaching hospitals in Tenerife and Zaragoza			symptoms for at least 3 days and that required treatment with antibiotics, and/or corticosteroids
Jones et al[19]	Devon Primary Care COPD audit data set	375 patients in primary care with confirmed COPD, of whom 197 (53%) had GOLD (Global Initiative for Chronic Obstructive Lung Disease) stage II disease, 144 (38%) had GOLD stage III disease, and 34 (9%) had GOLD stage IV disease.	D	Exacerbation	NA
Jones et al[19]	Devon Primary Care COPD audit data set	375 patients in primary care with confirmed COPD, of whom 197 (53%) had GOLD (Global Initiative for Chronic Obstructive Lung Disease) stage II disease, 144 (38%) had GOLD stage III disease, and 34 (9%) had GOLD stage IV disease.	IV	Exacerbation	NA
Jones et al[19]	Tokyo	diagnosis of COPD according to the ATS/ERS guidelines[18] and a history of smoking, who consulted the outpatient clinic of the Respiratory Care Clinic, Nippon Medical School, Tokyo, from January to April 2004 for ambulatory treatment in a stable condition.	EV	Exacerbation	NA
Jones et al[19]	Holland	One hundred and fifty four primary care patients diagnosed with COPD in the Holland. Patient data were gathered as part of a controlled clinical trial on the longterm effects of a multidisciplinary disease management programme on quality of life.	EV	Exacerbation	NA

Jones et al[19]	London	na	EV	Exacerbation	NA
Motegi et al[20]	Nippon Medical School	232 outpatients with COPD who were initially seen in the outpatient Respiratory Care Clinic (RCC), a secondary care specialty COPD clinic of Nippon Medical School, Tokyo, Japan, from April 2007 to October 2007	EV	Exacerbation	onset or worsening of more than two symptoms (dyspnea, sputum purulence or volume, cough, or wheeze) for more than 2 consecutive days
Ko et al[21]	Prince of Wales Hospital	prospective study of patients who had been admitted to the Prince of Wales Hospital with acute exacerbations of COPD (AECOPD) between May 1, 2004, and April 30, 2005.	EV	Exacerbation	Na
Moberg et al [22]	Hvidovre Hospital, Copenhagen	All patients included in the present analyses participated in a 7-week pulmonary rehabilitation program at Hvidovre Hospital, Copenhagen, during the period from March 2002 to March 2011.	EV	Exacerbation	primary diagnosis of COPD (J44.x), or a primary diagnosis of respiratory failure (J96.x) with a secondary diagnosis of COPD (J44.x) at discharge was recorded as an admission due to exacerbation in COPD

Li et al[23]	Beijing Municipal Health Big Data and Policy Research Center	retrospective cohort study based on the hospital discharge database maintained by the Beijing Municipal Health Big Data and Policy Research Center	D / IV	Exacerbation	NA
Wang et al[24]	First Affiliated Hospital of Guangzhou Medical University	334 participants were recruited from August 7, 2017, to February 15, 2022. All 334 participants performed the PFT and underwent chest CT scans for the first year. Then, these 334 participants took questionnaires every subsequent year	D	Exacerbation	NA
Chen et al[25]	National Taiwan University Hospital	participants were spirometry-confirmed COPD patients who were enrolled in the Taiwan nationwide COPD pay-for-performance program	D / IV	Exacerbation	severe acute exacerbation of COPD was defined as a disease status that necessitates emergency department visits or hospitalization.
Singh et al[26]	ETHOS, KRONOS, TELOS, SOPHOS, and PINNACLE-1, PINNACLE-2, and PINNACLE-4	The database included pooled exacerbation data from a total of 20,054 patients from ETHOS (NCT02465567), 13 KRONOS (NCT02497001), 12 TELOS (NCT02766608), 20 SOPHOS (NCT02727660), 21 PINNACLE-1 (NCT01854645), 22 PINNACLE-2 (NCT01854658), 22 and PINNACLE-4 (NCT02343458) 24 (Table 1). Data from extension studies (up to 1 year in duration) of KRONOS (NCT03262012, NCT02536508)18,19 and PINNACLE-1 and PINNACLE-2 (PINNACLE-3; NCT01970878) 23 were also included	D / IV	Exacerbation	NA
Huang et al[27]	Chongqing, China	new admission number of COPD patient per week were acquired from 2015 to 2018 in ten grade A tertiary hospitals in Chongqing, China. The responding PM2.5,	D / IV	Exacerbation	NA

		SO2, NO2 and CO values were collected from the website of China National Environmental Monitoring Center			
Crutsen et al[28]	Home Sweet Home	data from the "Home Sweet Home" study [27], a prospective, longitudinal observational study on the home environment of patients with COPD Global Initiative for Chronic Obstructive Lung Disease classification (GOLD-class) II, III, or IV.	D	Exacerbation	NA
Chen et al[29]	SPIROMICS	SPIROMICS is a multicenter prospective cohort study, enrolling participants from 2010 to 2015 at six clinical centers with a total of 11 recruitment locations.	D / IV	Exacerbation	report of worsening respiratory symptoms needing additional therapy
Yii et al[30]	COPD Registry of Changi General Hospital, Singapore,	data from the Changi General Hospital, Singapore COPD Registry for this study. The registry recruited consecutive patients aged ≥40 years, who attended the specialist COPD clinic at Changi General Hospital between January 1, 2008 and January 31, 2018.	D / IV	Exacerbation	primary or secondary discharge diagnosis of acute COPD exacerbation
Lovelace et al[31]	ECLIPSE	ECLIPSE recruited 2501 current and former smokers (COPD patients and controls), aged 45–80 with smoking history >10 pack-years in US and Europe (December 2005 to November 2007). We selected individuals with 3-year mortality data and no missing values among our selected features (n = 2312)	D / EV	Mortality	NA
Shah et al[32]	UK	CPRD dataset of 37,485 COPD patients that fulfilled the eligibility criteria and were included for subsequent analysis.	D / IV	Mortality	NA

Strand et al[32]	COPDGene; Gold 0-4 and PRISm	COPDGene Study recruited 10,198 non-Hispanic whites and African Americans aged 45-80 years at 21 clinical centers across the United States.	D / EV	Mortality	NA
Strand et al[32]	SPIROMICS; Gold 1-4	A second independent cohort from the SPIROMICS study was used for validation of the risk score model. Study design and enrollment of participants were similar to COPDGene and began in 2010.	D / EV	Mortality	NA
Dong et al al[32]	Beijing, China	retrospective study in National Clinical Research Center for Respiratory Diseases, a 354-bed, medical and clinical research center in a tertiary hospital in Beijing, China. Eligible patients were individuals who were diagnosed as AECOPD, aged ≥40 years and admitted between January 1, 2015 and December 31, 2019	D / IV / EV	Mortality	NA
Huang et al[33]	Sichuan, China	retrospective, observational, cohort study in the ICU with over 200 beds in a large tertiary-care teaching hospital in Chengdu city, Sichuan province, China.	D / IV	Mortality	NA
Pellicori et al[34]	Glasgow, Scotland	All admissions (SMR01 records) to hospitals served by NHS Greater Glasgow & Clyde with an ICD10 discharge code for COPD (J440, J441, J448, J449) in any diagnostic position, between 01/01/2010 and 31/12/2017 were identified.	D	Mortality	NA
Owusuaa et al[35]	Netherlands	prospectively included patients from the Pulmonary Diseases departments and outpatient clinics in five hospitals in the Netherlands: two general (Van Weel Bethesda Hospital and Admiraal De Ruyter Hospital) and three teaching hospitals (Amphia Hospital, Maasstad Hospital, and Ikazia Hospital).	D / IV / EV	Mortality	NA

Bloom et al[36]	UK	CPRD dataset. Two study populations were drawn. The first had a cohort start date of 1 January 2010, and an arbitrary index date. A second population was drawn that did not have a recorded annual review date and had data drawn from an earlier time period.	D / IV / EV	Mortality	NA
Tsimogianni et al[37]	Athens, Greece	All consecutive patients over the age of 40 years with a diagnosis of COPD and symptoms indicative of an exacerbation who were admitted at a respiratory department of "Sotiria" Chest Diseases Hospital in Athens, Greece, from July 2001 to June 2004,	D / EV	Mortality	NA
Sun et al[38]	Wuhu, China	retrospective clinical study involved a total of 1224 hospitalized patients who were diagnosed with AECOPD at the Second People's Hospital of Wuhu City between January 2013 and December 2022.	D / IV	Mortality	NA
Horne et al[39]	SUMMIT cohort	subjects from the SUMMIT trial who were individuals ages 40–80 years with COPD who had moderate airflow limitation and a history of or elevated risk of cardiovascular disease	D	Mortality	NA
Horne et al[39]	SUMMIT cohort	subjects from the SUMMIT trial who were individuals ages 40–80 years with COPD who had moderate airflow limitation and a history of or elevated risk of cardiovascular disease	IV / EV	Mortality	NA
Horne et al[39]	Intermountain Cardiovascular	Intermountain outpatients with elevated cardiovascular risks (N=9251) were ages 40–80 years with a history of CAD, MI, stroke, peripheral arterial disease, or diabetes, or ages 60–80 years who were receiving treatments for one or more of the following: hypercholesterolemia,	EV	Mortality	NA

		hypertension, or vascular disease, approximating the cardiovascular risk definition from SUMMIT			
Liao et al[40]	Taiwan	retrospective study collected the data of patients with COPD (pulmonary obstruction) with either emergency, outpatient, or inpatient orders from the three hospitals of Chi Mei Medical Group in Taiwan (1 medical center, 1 regional hospital, and 1 district hospital), from 1 January 2010 to 31 December 2019,	D / IV	Mortality	NA
Ryynänen et al[41]	Finland	Hospital Discharge Registries were used to identify all patients with COPD who had visited the Pulmonary Clinics of the Helsinki and Turku University Hospitals	D / IV	Mortality	NA

AF – atrial fibrillation; ATS – American Thoracic Society; CAD – coronary artery disease; CanCOLD - Canadian Cohort Obstructive Lung Disease; COPD – chronic obstructive pulmonary disease; CT -Computed Tomography; CPRD – clinical practice research database; CVD – cardiovascular disease; ED – Emergency Department; ERS – European Respiratory Society; ECLIPSE - Evaluation of COPD Longitudinally to Identify Predictive Surrogate End-points; ETHOS - Efficacy and Safety of Triple Therapy in Obstructive Lung Disease; ICD – International Classification of Diseases; MI – myocardial infarction; NHANES - National Health and Nutrition Examination Survey; PFT – pulmonary function test; PRISm – preserved ratio impaired spirometry; SPIROMICS - SubPopulations and InteRmediate Outcome Measures in COPD Study; SUMMIT – Study to understand mortality and morbidity in COPD; TORCH - TOwards a Revolution in COPD Health; USA – United States of America;

Supplementary Table 4: Demographic and environmental variables used in the models

Model name	Age	Sex	Weight	BMI	Smoking	Insurance Coverage	Alcohol	Socio-economic status	temperature	humidity	fine particulate matter	SO2	NO2	CO	Calorie intake
Clinical CT-based whole lung radiomics nomogram[7]	X		X												
Combined CT-based whole lung radiomics nomogram[7]	X		X												
A nomogram to predict the occurence of AF in COPD patients[8]	X														
A nomogram to predict the occurence of CVD in COPD patients[9]	X	X		X	X										
multivariable logistic regression (Shi)[10]	X	X		X											
random forest model (Shi) [10]	X	X		X	X			X							
Conditional model[11]	X	X		X	X										

ACCEPT 1.0 (multivariate predictive model)[12]	X	X		X	X										
ACCEPT 2.0(multivariable predictive model) [13]	X	X		X	X										
Samp model [14]	X	X				X									
Singla CT-base deep learning model direct [15]															
Singla CT-base deep learning model indirect [15]															
Random Forest Model [16]									X	X	X				X
Decision trees [16]									X	X	X				X
k-nearest neighbor [16]									X	X	X				X
LDA[16]									X	X	X				X
AdaBoost[16]									X	X	X				X
Deep Neural Network[16]									X	X	X				X
Bertens [17]					X										
ADO-SQ[35]	X														
Auto-Metric Graph Neural Network (AMGNN)[24]															
BARC[37]	X	X		X	X										
BHDE[25]				X											
BMI[37]				X											
BODE[18 20 31]				X											
BODEu[31]				X											
BODEX[35 36]				X											

BOSE-6wk[21]				X											
Chen Nomogram[29]				X											
CODEX[35]															
Crutsen Model[28]		X			X										
Dong Model[42]	X														
DOSE[19 20 35]					X										
Huang LWLR											X	X	X	X	
Huang Model[33]	X														
iBODE[22]				X											
Li Model [23]	X	X													
Pellicori Model[34]	X	X													
Simplified Score				X											
Singh-Darken negative binomial GLM Full[26]		X													
ADO-SQ[35]	X														
Strand Full, 3-level Emphysema[43]	X			X	X										
Strand Full, Binary Emphysema Model[43]	X			X	X										
Strand Reduced Model[43]	X			X	X										
Summit Score[39]	X			X	X										
Sun Model A[38]															
Sun Model B[38]															
Support Vector Machines (SVM)[24]															
Shah[32]	X	X		X	X			X							
Updated BODE[44]				X											
VAPORED	X														
XGBoost Model[40]	X	X		X											
Bayesian Predictors[41]				X			X								

ADO-SQ – age, dyspnoea, airflow obstruction, surprise question; AF – atrial fibrillation; BARC - Body mass index, Age, Respiratory variables, and Comorbidities; BHDE - Body mass index, Heart rate recovery, Dyspnea score, and Exercise capacity; BMI – body mass index; BODE - Body mass index, airflow Obstruction, Dyspnea score, and Exercise capacity; BODEx – BODE and exacerbations; CODEX - Charlson comorbidity score (age-adjusted), Obstruction (FEV1 or forced expiratory volume in one second), Dyspnea (shortness of breath), and severe Exacerbations; CO2 – carbon dioxide; CT -Computed Tomography; CO – carbon monoxide; COPD – chronic obstructive pulmonary disease; DOSE - Dyspnea, Obstruction, Smoking, Exacerbation; NO2 – nitrogen dioxide; VAPORED - Vital capacity-FVC %predicted, Age, history of Pneumonia, Oxygen saturation, the FEV1/FVC Ratio, 6-min walk Exercise capacity, Dyspnea

Supplementary Table 5: Respiratory variables used in the models

Model name	LTOT	NIV	Level of Obstr-uction	Inhaler Treatm-ent	sO2	RR	Asthma	Lung Cancer	Lung Fibrosis	Respiratory Failure	ALF	Pneumothorax	Acute Resp Infections	Sputum NTM isolation	Exacerbation History	FEV1	FVC	FEV1 /FVC	Bronchiectasis
Conditional model[11]	X			X											X	X			
ACCEPT 1.0 (multivariate predictive model)[12]	X			X												X			
ACCEPT 2.0(multivariable predictive model) [13]	X															X			
Samp[14]				X															
Bertens[17]			X												X				
ADO[31 35 36]																X			
ADO-SQ[35]																X			
BARC[37]							X	X	X						X	X			
BODE[18 20 31]																X			
BODEu[31]																X			
BODEX[35 36]															X	X			
BOSE-6wk[21]																X			
Chen Nomogram [29]															X	X			
CODEX[35]															X	X			
Crutsen Model[28]															X		X		
Dong Model[42]										X		X							
DOSE[19 20 35]															X	X			
FEVX																X			
iBODE[22]																X			
Li Model [23]							X								X				

Pellicori Model[34]	X																		
Singh-Darken negative binomial GLM Full[26]				X											X	X			
Singh-Darken negative binomial GLM Simplified[26]				X											X				
Strand Full, 3-level Emphysema[43]																X			
Strand Full, Binary Emphysema Model[43]																X			
Strand Reduced Model[43]															X	X			
Summit Score[39]								X			X								
Sun Model A[38]								X											
Support Vector Machines (SVM)																			
Shah[32]													X		X				
Updated BODE[44]																X			
VAPORED					X								X				X	X	
XGBoost Model[40]					X	X							X						
Yii "Treatable Traits"[30]		X												X	X				X
Bayesian Predictors[41]							X	X								X			

ADO-SQ – age, dyspnoea, airflow obstruction, surprise question; AF – atrial fibrillation; ALF – acute lung failure; BARC - Body mass index, Age, Respiratory variables, and Comorbidities; BHDE - Body mass index, Heart rate recovery, Dyspnea score, and Exercise capacity; BMI – body mass index; BODE - Body mass index, airflow Obstruction, Dyspnea score, and Exercise capacity; BODEx – BODE and exacerbations; CODEX - Charlson comorbidity score (age-adjusted), Obstruction (FEV1 or forced expiratory volume in one second), Dyspnea (shortness of breath), and severe Exacerbations; CO2 – carbon dioxide; CT -Computed Tomography; CO – carbon monoxide; COPD – chronic obstructive pulmonary disease; DOSE - Dyspnea, Obstruction, Smoking, Exacerbation; FEV1 – forced expiratory volume in 1 second; FVC – forced vital capacity; LTOT – long term oxygen therapy; NIV – non invasive ventilation; NTM – non tuberculous mycobacteria; NO2 – nitrogen dioxide; RR – respiratory rate; sO2 – oxygen saturation; VAPORED - Vital capacity-FVC %predicted, Age, history of Pneumonia, Oxygen saturation, the FEV1/FVC Ratio, 6-min walk Exercise capacity, Dyspnea

Supplementary Table 6: Cardiovascular variables used in the models

Model name	HTN	IHD	HF	MI	AF	QTc	DM	Stroke	TIA	Cerebrovascular Disease	CCF	CVD	SBP	DBP	Heart rate	Total Cholesterol	HDL Cholesterol	FHx of heart disease	Left atrial diameter
A nomogram to predict the occurence of AF in COPD patients[8]																			X
A nomogram to predict the occurence of CVD in COPD patients[9]																		X	
multivariable logistic regression[10]																	X	X	
Random Forest Model (Shi) [16]													X	X			X		
Conditional model[11]	X	X		X			X												
Random Forest Model (Wu) [16]																X			
Decision trees [16]																X			
k-nearest neighbor [16]																X			
LDA[16]																X			
AdaBoost[16]																X			
Deep Neural Network[16]																X			
Bertens[17]												X							
BARC[37]					X			X											
BHDE[25]															X				
Strand Model[43]							X						X						
Li Model[23]	X	X	X				X			X									
Strand Full, 3-level Emphysema[43]							X					X							
Strand Full, Binary							X					X							

Emphysema Model[43]																			
Strand Reduced Model[43]							X					X							
Summit Score[39]			X	X			X						X		X				
Sun Model A[38]															X				
Sun Model B[38]															X				
Shah[32]	X		X																
XGBoost Model[40]	X						X	X	X		X								
Yii "Treatable Traits"[30]		X																	
Bayesian Predictors[41]					X	X	X			X									

ADO-SQ – age, dyspnoea, airflow obstruction, surprise question; AF – atrial fibrillation; ALF – acute lung failure; BARC - Body mass index, Age, Respiratory variables, and Comorbidities; BHDE - Body mass index, Heart rate recovery, Dyspnea score, and Exercise capacity; BMI – body mass index; BODE - Body mass index, airflow Obstruction, Dyspnea score, and Exercise capacity; BODEx – BODE and exacerbations; ; CCF – congestive cardiac failure; CODEX - Charlson comorbidity score (age-adjusted), Obstruction (FEV1 or forced expiratory volume in one second), Dyspnea (shortness of breath), and severe Exacerbations; CO2 – carbon dioxide; CT -Computed Tomography; CO – carbon monoxide; COPD – chronic obstructive pulmonary disease; DOSE - Dyspnea, Obstruction, Smoking, Exacerbation; DBP – diastolic blood pressure; DM – diabetes mellitus; FHx – family history; HDL – high density lipoprotein; HF – heart failure; HTN – hypertension; IHD – ischaemic heart disease; FEV1 – forced expiratory volume in 1 second; FVC – forced vital capacity; LTOT – long term oxygen therapy; NIV – non invasive ventilation; NTM – non tuberculous mycobacteria; NO2 – nitrogen dioxide; RR – respiratory rate; sO2 – oxygen saturation; VAPORED - Vital capacity-FVC %predicted, Age, history of Pneumonia, Oxygen saturation, the FEV1/FVC Ratio, 6-min walk Exercise capacity, Dyspnea; SBP – systolic blood pressure; TIA – transient ischaemic attack;

Supplementary Table 7: Biochemical and imaging variables used in the models

Model name	BUN	Renal function	Hb	Albumin	CRP	HbA1c	Fibrinogen	IL6	PCT	DDimer	Serum chloride	uric acid	WCC	NEU	PLT	LYM	EOS	Exercise	6MWT	ISWT	TUG-time
Random Forest Model (Wu) [16]																		X			
Decision trees [16]																		X			
k-nearest neighbor [16]																		X			
LDA[16]																		X			
AdaBoost[16]																		X			
Deep Neural Network[16]																		X			
BARC[37]		X	X	X	X										X						
BHDE[25]																			X		
BODE[18 20 31]																			X		
BODEu[31]																			X		
BOSE-6wk[21]																			X		
CODEX[35]																					
DOSE[19 20 35]																					
Strand Model[43]	X						X	X													
iBODE[22]																				X	
Pellicori Model[34]	X	X		X										X		X					
Singh-Darken negative binomial GLM Full[26]																	X				
Strand Full, 3-level Emphysema[43]																			X		
Strand Full, Binary Emphysema Model[43]																			X		
Sun Model A[38]	X		X	X				X	X	X	X			X							
Sun Model B[38]	X		X	X						X	X			X							
Support Vector Machines (SVM)[24]																					
Shah[32]																		X			
Updated BODE[44]																			X		
VAPORED																			X		
XGBoost Model[40]	X	X											X		X						

BUN – blood urea nitrogen; CRP – c reactive protein; EOS – eosinophils; Hb – haemoglobin; IL6 – Interleukin 6; ISWT – incremental shuttle walking test; LYM – lymphocytes; neutrophils; PCT – procalcitonin; PLT – platelets; TUG-time – time to get up and go time; WCC – white cell count; 6MWT – 6 minute walk test; ADO-SQ – age, dyspnoea, airflow obstruction, surprise question; AF – atrial fibrillation; ALF – acute lung failure; BARC - Body mass index, Age, Respiratory variables, and Comorbidities; BHDE - Body mass index, Heart rate recovery, Dyspnea score, and Exercise capacity; BMI – body mass index; BODE - Body mass index, airflow Obstruction, Dyspnea score, and Exercise capacity; BODEx – BODE and exacerbations; ; CCF – congestive cardiac failure; CODEX - Charlson comorbidity score (age-adjusted), Obstruction (FEV1 or forced expiratory volume in one second), Dyspnea (shortness of breath), and severe Exacerbations; CO2 –

carbon dioxide; CT -Computed Tomography; CO – carbon monoxide; COPD – chronic obstructive pulmonary disease; DOSE - Dyspnea, Obstruction, Smoking, Exacerbation; DBP – diastolic blood pressure; DM – diabetes mellitus; FHx – family history; HDL – high density lipoprotein; HF – heart failure; HTN – hypertension; IHD – ischaemic heart disease; FEV1 – forced expiratory volume in 1 second; FVC – forced vital capacity; LTOT – long term oxygen therapy; NIV – non invasive ventilation; NTM – non tuberculous mycobacteria; NO2 – nitrogen dioxide; RR – respiratory rate; sO2 – oxygen saturation; VAPORED - Vital capacity-FVC %predicted, Age, history of Pneumonia, Oxygen saturation, the FEV1/FVC Ratio, 6-min walk Exercise capacity, Dyspnea; SBP – systolic blood pressure; TIA – transient ischaemic attack;

Supplementary Table 8: Miscellaneous variables used in prediction models

Model name	Rapid eye movement	Antithrombotics	Antiarrhythmics	Xanthines	LOS	Blood transfusion	Comorbidity	Depression	Anxiety	Psychiatric Disease	CKD	Dementia	Cancer
A nomogram to predict the occurence of CVD in COPD patients[9]						X							
Conditional model[11]													X
Samp model[14]							X						
Random Forest Model (Wu) [16]	X												
Decision trees [16]	X												
k-nearest neighbor [16]	X												
LDA[16]	X												
AdaBoost[16]	X												
Deep Neural Network[16]	X												
BARC[37]											X		
Chen Nomogram[29]							X						
Dong Model[42]					X								
Strand Model[43]											X		
Pellicori Model[34]					X							X	X
Strand Full, 3-level Emphysema[43]													X
Strand Full, Binary Emphysema Model[43]													X
Strand Reduced Model[43]													X
Summit Score[39]		X	X	X									
Shah[32]								X	X				
Bayesian Predictors[41]										X			

CKD – chronic kidney disease; CVD – cardiovascular disease; LOS – length of stay;

Supplementary Table 9: Indices used in prediction models

Model name	Charlson Comorbidity	GOLD	radiomics/HRCT	CAT score	Surprise Question	mMRC Dyspnoea scale	St. George's Respiratory Questionnaire score	CIROPD%correct	CDS	MOSSSS
Clinical CT-based whole lung radiomics nomogram[7]		X								
Radiomics-only CT-based whole lung radiomics nomogram[7]			X							
Combined CT-based whole lung radiomics nomogram[7]		X	X							
Conditional model[11]						X				
ACCEPT 1.0 (multivariate predictive model)[12]							X			
Singla CT-base deep learning model direct[15]						X				
ADO[31 35 36]						X				
ADO-SQ[35]					X	X				
Auto-Metric Graph Neural Network (AMGNN) [24]			X							
BARC[37]						X				
BHDE[25]						X				
BODE[18 20 31]						X				
BODEX[35 36]						X				
BOSE-6wk[21]						X				
CODEX[35]						X				
Crutsen Model[28]								X	X	X
DOSE[19 20 35]						X				
iBODE[22]						X				
Li Model [23]	X									
Linear Discriminant Analysis (LDA)[24]			X							
MRC[37]						X				
Multilayer Perceptron (MLP) [24]			X							
Random Forest Model[24]			X							
Simple Neural Attentive Learner (SNAIL) [24]			X							

Simplified Score[35]						X				
Singh-Darken negative binomial GLM Full[26]				X						
Strand Full, 3-level Emphysema[43]			X			X				
Strand Full, Binary Emphysema Model[43]			X			X				
Strand Reduced Model[43]						X				
Support Vector Machines (SVM)			X							
Updated BODE[44]						X				
VAPORED						X				
XGBoost Model[40]						X				

CAT – COPD assessment test ; CDS – care dependency scale; $CIROPD_{\%correct}$ - COPD knowledge questionnaire, percentage correctly answered questions; GOLD – Global initiative for chronic obstructive lung disease; HRCT – high resolution computed tomography; MOSSS - Medical Outcome Study Social Support Survey; BUN – blood urea nitrogen; CRP – c reactive protein; EOS – eosinophils; Hb – haemoglobin; IL6 – Interleukin 6; ISWT – incremental shuttle walking test; LYM – lymphocytes; neutrophils; PCT – procalcitonin; PLT – platelets; TUG-time – time to get up and go time; WCC – white cell count; 6MWT – 6 minute walk test; ADO-SQ – age, dyspnoea, airflow obstruction, surprise question; AF – atrial fibrillation; ALF – acute lung failure; BARC - Body mass index, Age, Respiratory variables, and Comorbidities; BHDE - Body mass index, Heart rate recovery, Dyspnea score, and Exercise capacity; BMI – body mass index; BODE - Body mass index, airflow Obstruction, Dyspnea score, and Exercise capacity; BODEx – BODE and exacerbations; ; CCF – congestive cardiac failure; CODEX - Charlson comorbidity score (age-adjusted), Obstruction (FEV1 or forced expiratory volume in one second), Dyspnea (shortness of breath), and severe Exacerbations; CO2 – carbon dioxide; CT -Computed Tomography; CO – carbon monoxide; COPD – chronic obstructive pulmonary disease; DOSE - Dyspnea, Obstruction, Smoking, Exacerbation; DBP – diastolic blood pressure; DM – diabetes mellitus; FHx – family history; HDL – high density lipoprotein; HF – heart failure; HTN – hypertension; IHD – ischaemic heart disease; FEV1 – forced expiratory volume in 1 second; FVC – forced vital capacity; LTOT – long term oxygen therapy; NIV – non invasive ventilation; NTM – non tuberculous mycobacteria; NO2 – nitrogen dioxide; RR – respiratory rate; sO2 – oxygen saturation; VAPORED - Vital capacity-FVC %predicted, Age, history of Pneumonia, Oxygen saturation, the FEV1/FVC Ratio, 6-min walk Exercise capacity, Dyspnea; SBP – systolic blood pressure; TIA – transient ischaemic attack; MRC – medical research council

Supplementary Table 10: Performance metrics of prediction models for cardiovascular events

Author	Aim	Model	c-index/AUC	Lower 95% CI	Upper 95% CI	Prediction horizon of model (years)
Krishnan et al[6]	EV	Framingham Risk Score	0.64	0.55	0.74	6.3
Krishnan et al[6]	EV	Pooled Cohort Equations (PCE)	0.57	0.41	0.73	6.3
Lin et al[7]	D	Clinical CT-based whole lung radiomics nomogram	0.61	0.546	0.663	5
Lin et al[7]	IV	Clinical CT-based whole lung radiomics nomogram	0.63	0.543	0.715	5
Lin et al[7]	EV	Clinical CT-based whole lung radiomics nomogram	0.69	0.639	0.741	5
Lin et al[7]	D	Radiomics-only CT-based whole lung radiomics nomogram	0.73	0.677	0.778	5
Lin et al[7]	IV	Radiomics-only CT-based whole lung radiomics nomogram	0.72	0.633	0.801	5
Lin et al[7]	EV	Radiomics-only CT-based whole lung radiomics nomogram	0.7	0.648	0.756	5
Lin et al[7]	D	Combined CT-based whole lung radiomics nomogram	0.73	0.68	0.782	5
Lin et al[7]	IV	Combined CT-based whole lung radiomics nomogram	0.73	0.645	0.81	5
Lin et al[7]	EV	Combined CT-based whole lung radiomics nomogram	0.73	0.674	0.777	5
Huang et al[8]	D/IV	A nomogram to predict the occurrence of AF in COPD patients	0.89	0.842	0.93	3
Qu et al[9]	D	A nomogram to predict the occurrence of CVD in COPD patients	0.75	0.71	0.79	na
Qu et al[9]	D	A nomogram to predict the occurrence of CVD in COPD patients	0.79	0.73	0.85	na
Shi et al[10]	IV	multivariable logistic regression	0.74	na	na	na
Shi et al[10]	IV	random forest model	0.98	na	na	na

AF – atrial fibrillation; COPD – chronic obstructive pulmonary disease; CT – computed tomography; CVD – cardiovascular disease; D – Derivation; EV – External Validation; IV – Internal Validation

Supplementary Table 11: Performance metrics of prediction models for exacerbations

Author	Aim	Model	c-index/AUC	Lower 95% CI	Upper 95% CI	Prediction horizon of model (years)
Hussein et al[11]	IV	Conditional model	0.76	0.72	0.79	2
Hussein et al[11]	D	Conditional model	0.88	0.77	0.99	2
Adibi et al[12]	D	ACCEPT 1.0 (multivariate predictive model)	NA	NA	NA	NA
Adibi et al[12]	EV	ACCEPT 1.0 (multivariate predictive model)	0.77	0.74	0.8	NA
Safari et al[13]	EV	ACCEPT 2.0(multivariable predictive model)	0.76	0.72	0.79	NA
Samp et al[14]	D	Samp Model	0.71	na	na	NA
Singla et al[15]	D	Singla CT-base deep learnig model direct	0.68	0.64	0.72	NA
Singla et al[15]	IV	Singla CT-base deep learnig model indirect	0.73	0.71	0.75	NA
Wu et al[16]	D/IV	Random Forest Model	0.99	NA	NA	NA
Wu et al[16]	D/IV	Decision trees	0.8	NA	NA	NA
Wu et al[16]	D/IV	k-nearest neighbor	0.78	NA	NA	NA
Wu et al[16]	D/IV	LDA	0.88	NA	NA	NA
Wu et al[16]	D/IV	AdaBoost	0.97	NA	NA	NA
Wu et al[16]	D/IV	Deep Neural Network	0.96	NA	NA	NA
Bertens et al[17]	D	Bertens	0.75	0.69	0.82	NA
Bertens et al[17]	IV	Bertens	0.66	0.61	0.71	NA
Marin et al[18]	EV	BODE	0.81	0.75	0.87	8
Jones et al[19]	D	DOSE	NA	NA	NA	1
Jones et al[19]	IV	DOSE	NA	NA	NA	1
Jones et al[19]	EV	DOSE	NA	NA	NA	1
Jones et al[19]	EV	DOSE	NA	NA	NA	1
Jones et al[19]	EV	DOSE	0.76	NA	NA	1
Motegi et al[20]	EV	BODE	0.65	0.56	0.73	8
Motegi et al[20]	EV	DOSE	0.75	0.67	0.82	1
Motegi et al[20]	EV	ADO	0.64	0.56	0.73	8
Ko et al[21]	EV	BOSE-6wk	0.58	NA	NA	3
Moberg et al[22]	EV	iBODE	NA	NA	NA	5
Li et al[23]	D	Li Model	0.79	0.779	0.792	1
Li et al[23]	IV	Li Model	0.78	0.77	0.79	1
Wang et al[24]	D	Random Forest Model	0.8	0.75888	0.84512	1
Wang et al[24]	D	Multilayer Perceptron (MLP)	0.92	0.86804	0.96996	1
Wang et al[24]	D	Linear Discriminant Analysis (LDA)	0.93	0.8948	0.9732	1
Wang et al[24]	D	Support Vector Machines (SVM)	0.92	0.9014	0.9406	1
Wang et al[24]	D	Simple Neural Attentive Learner (SNAIL)	0.94	0.89292	0.98308	1

Wang et al[24]	D	Auto-Metric Graph Neural Network (AMGNN)	0.95	0.91668	0.98332	1
Chen et al[29]	D	BHDE	0.76	NA	NA	1
Chen et al[29]	IV	BHDE	0.74	NA	NA	1
Singh et al[26]	D	Singh-Darken negative binomial GLM Full	0.7	NA	NA	1
Singh et al[26]	IV	Singh-Darken negative binomial GLM Full	0.71	NA	NA	1
Singh et al[26]	D	Singh-Darken negative binomial GLM Simplified	0.67	NA	NA	1
Singh et al[26]	IV	Singh-Darken negative binomial GLM Simplified	0.65	NA	NA	1
Huang et al[27]	D	Huang LWLR	NA	NA	NA	NA
Huang et al[27]	IV	Huang LWLR	NA	NA	NA	NA
Crutsen et al[28]	D	Crutsen Model	0.86	0.794	0.927	1
Chen et al[29]	D	Chen Nomogram	0.74	0.71	0.76	3
Chen et al[29]	IV	Chen Nomogram	NA	NA	NA	3
Chen et al[29]	D	Chen Nomogram	0.74	0.71	0.76	5
Chen et al[29]	IV	Chen Nomogram	NA	NA	NA	5
Yi et al[30]	D	Yii "Treatable Traits"	0.79	0.732	0.846	1
Yi et al[30]	IV	Yii "Treatable Traits"	0.73	0.68	0.777	1

ADO-SQ – age, dyspnoea, airflow obstruction, surprise question; BHDE - Body mass index, Heart rate recovery, Dyspnea score, and Exercise capacity; BODE - Body mass index, airflow Obstruction, Dyspnea score, and Exercise capacity; BODEx – BODE and exacerbations; D – Derivation; EV – External Validation; IV – Internal Validation

Supplementary Table 12: Performance metrics of prediction models for all-cause mortality

Author	Aim	Model	c-index/AUC	Lower 95% CI	Upper 95% CI	Prediction horizon of model (years)
Lovelace et al[31]	EV	VAPORED	0.73	NA	NA	3
Lovelace et al[31]	EV	ADO	0.7	NA	NA	3
Lovelace et al[31]	EV	BODEu	0.7	NA	NA	3
Lovelace et al[31]	EV	BODE	0.7	NA	NA	3
Lovelace et al[31]	D	VAPORED	NA	NA	NA	10
Shah et al[32]	IV	TRIPOD	0.80-0.83	NA	NA	10
Shah et al[32]	D	TRIPOD	0.78-0.85	NA	NA	10
Strand et al[43]	D	Strand Full, Binary Emphysema Model	0.8	NA	NA	6
Strand et al[43]	D	Strand Full, 3-level Emphysema	0.8	NA	NA	6
Strand et al[43]	EV	BODE	0.78	NA	NA	6
Strand et al[43]	D	Strand Reduced Model	0.78	NA	NA	6
Strand et al[43]	D	Strand Full, Binary Emphysema Model	0.79	NA	NA	6
Strand et al[43]	D	Strand Full, 3-level Emphysema	0.79	NA	NA	6
Strand et al[43]	EV	BODE	0.77	NA	NA	6
Strand et al[43]	D	Strand Reduced Model	0.78	NA	NA	6
Strand et al[43]	EV	Strand Full, Binary Emphysema Model	0.76	NA	NA	6
Strand et al[43]	EV	Strand Reduced Model	0.71	NA	NA	6
Strand et al[43]	EV	BODE	0.69	NA	NA	6
Strand et al[43]	EV	Strand Full, Binary Emphysema Model	0.77	NA	NA	6
Strand et al[43]	EV	Strand Reduced Model	0.74	NA	NA	6
Strand et al[43]	EV	BODE	0.7	NA	NA	6
Dong et al[42]	D	Dong Model	NA	NA	NA	2
Dong et al[42]	IV	Dong Model	0.91	0.8859	0.9444	2
Dong et al[42]	EV	Dong Model	0.82	0.7487	0.8865	2
Huang et al[33]	D	Huang Model	0.84	0.809	0.872	0
Huang et al[33]	IV	Huang Model	0.83	0.781	0.878	0
Pellicori et al[34]	D	Pellicori Model	0.81	0.7917	0.82	0
Owusuaa et al[35]	D	ADO-SQ	0.79	0.73	0.85	1
Owusuaa et al[35]	IV/EV	ADO-SQ	0.83	0.73	0.93	1
Owusuaa et al[35]	IV/EV	ADO-SQ	0.78	0.71	0.86	1
Owusuaa et al[35]	IV/EV	ADO-SQ	0.55	0.31	0.78	1
Owusuaa et al[35]	IV/EV	ADO-SQ	0.82	0.69	0.96	1
Owusuaa et al[35]	EV	BODEX	0.71	0.65	0.77	1
Owusuaa et al[35]	EV	CODEX	0.68	0.61	0.75	1
Owusuaa et al[35]	EV	ADO	0.73	0.67	0.8	1
Owusuaa et al[35]	EV	SQ	0.7	0.63	0.76	1
Bloom et al[36]	D	BARC	NA	NA	NA	1
Bloom et al[36]	IV	BARC	0.78	0.764	0.792	1
Bloom et al[36]	IV	ADO	0.68	0.655	0.694	1
Bloom et al[36]	IV	BODEx	0.48	0.453	0.512	1
Bloom et al[36]	IV	DOSE	0.59	0.568	0.614	1

Bloom et al[36]	EV	BARC	0.7	0.671	0.719	1
Bloom et al[36]	EV	BODEx	0.41	0.379	0.447	1
Bloom et al[36]	EV	DOSE	0.52	0.485	0.546	1
Bloom et al[36]	EV	ADO	0.57	0.541	0.595	1
Tsimogianni et al[37]	EV	MRC	0.76	0.64	0.89	3
Tsimogianni et al[37]	EV	BMI	0.73	0.59	0.86	3
Tsimogianni et al[37]	D	Simplified Score	0.83	0.72	0.94	3
Tsimogianni et al[37]	EV	FEV1	0.7	0.56	0.83	3
Tsimogianni et al[37]	D	BOD Score	0.86	0.76	0.96	3
Sun et al[38]	D	Sun Model A	0.86	0.82	0.898	NA
Sun et al[38]	D	Sun Model B	0.86	0.742	0.827	NA
Sun et al[38]	IV	Sun Model B	0.85	0.805	0.893	NA
Horne et al[39]	D	Summit Score	0.67	NA	NA	2
Horne et al[39]	EV	ADO	0.59	0.567	0.617	2
Horne et al[39]	IV	Summit Score	0.66	NA	NA	2
Horne et al[39]	EV	ADO	0.62	0.593	0.644	2
Horne et al[39]	EV	Summit Score	0.74	NA	NA	6
Horne et al[39]	EV	Summit Score	NA	NA	NA	6
Horne et al[39]	EV	Summit Score	NA	NA	NA	4
Liao et al[40]	D	XGBoost Model	NA	NA	NA	NA
Liao et al[40]	IV	XGBoost Model	0.82	0.604	0.817	NA
Ryynänen et al[40]	D	PREQ	NA	NA	NA	1
Ryynänen et al[40]	IV	PREQ	0.69	NA	NA	1

ADO-SQ – age, dyspnoea, airflow obstruction, surprise question; BHDE - Body mass index, Heart rate recovery, Dyspnea score, and Exercise capacity; BODE - Body mass index, airflow Obstruction, Dyspnea score, and Exercise capacity; BODEx – BODE and exacerbations; VAPORED - Vital capacity-FVC %predicted, Age, history of Pneumonia, Oxygen saturation, the FEV1/FVC Ratio, 6-min walk Exercise capacity, Dyspnea; ; BARC - Body mass index, Age, Respiratory variables, and Comorbidities; DOSE - Dyspnea, Obstruction, Smoking, Exacerbation; MRC - medical research council; FEV1 – forced expiratory volume 1 second; D – Derivation; EV – External Validation; IV – Internal Validation

Supplementary Table 13: Risk of Bias and Applicability Assessment for Each PROBAST Domain for studies predicting cardiovascular events

Author	ROB Participants	Applicability Participants	ROB Predictors	Applicability Predictors	ROB Outcome	Applicability Outcomes	ROB Analysis	ROB Overall	Applicability Overall
Krishnan et al[6]									
Lin et al[7]									
Huang et al[8]									
Qu et al[9]									
Shi et al[10]									

Risk of bias and applicability represented with traffic light system. Red represents either high risk of bias or high concerns of applicability, yellow represents some concerns for risk of bias or some concerns for applicability, green represents low risk of bias or low concerns for applicability.

Supplementary Table 14: Risk of Bias and Applicability Assessment for Each PROBAST Domain for studies predicting exacerbations

Author	ROB Participants	ROB Predictors	ROB Outcome	ROB Analysis	Applicability Participants	Applicability Predictors	Applicability Outcomes	ROB Overall	Applicability Overall
Hussein et al [11]									
Adibi et al[12]									
Safari et al[13]									
Samp et al[14]									
Singla et al[15]									
Wu et al[16]									
Bertens et al[17]									
Marin et al[18]									
Jones et al[19]									
Motegi et al[20]									
Ko et al[21]									
Moberg et al[22]									
Li et al[23]									
Wang et al[24]									
Chen et al[25]									
Singh et al[26]									
Huang et al[27]									
Crutsen et al[28]									
Chen et al[29]									
Yi et al[30]									

Risk of bias and applicability represented with traffic light system. Red represents either high risk of bias or high concerns of applicability, yellow represents some concerns for risk of bias or some concerns for applicability, green represents low risk of bias or low concerns for applicability.

Supplementary Table 15: Risk of Bias and Applicability Assessment for Each PROBAST Domain for studies predicting all-cause mortality

**Author**	ROB Participants	Applicability Participants	ROB Predictors	Applicability Predictors	ROB Outcome	Applicability Outcomes	ROB Analysis	ROB Overall	Applicability Overall
Shah et al[32]									
Strand et al[43]									
Dong et al[42]									
Huang et al[33]									
Pellicori et al[34]									
Owusuaa et al[35]									
Lovelace et al[31]									
Bloom et al[36]									
Tsimogianni et al[37]									
Sun et al[38]									
Horne et al[39]									
Liao et al[40]									
Ryynänen et al[41]									

Risk of bias and applicability represented with traffic light system. Red represents either high risk of bias or high concerns of applicability, yellow represents some concerns for risk of bias or some concerns for applicability, green represents low risk of bias or low concerns for applicability.

Supplementary Figure 1: PRISMA Flow diagram demonstrating study selection

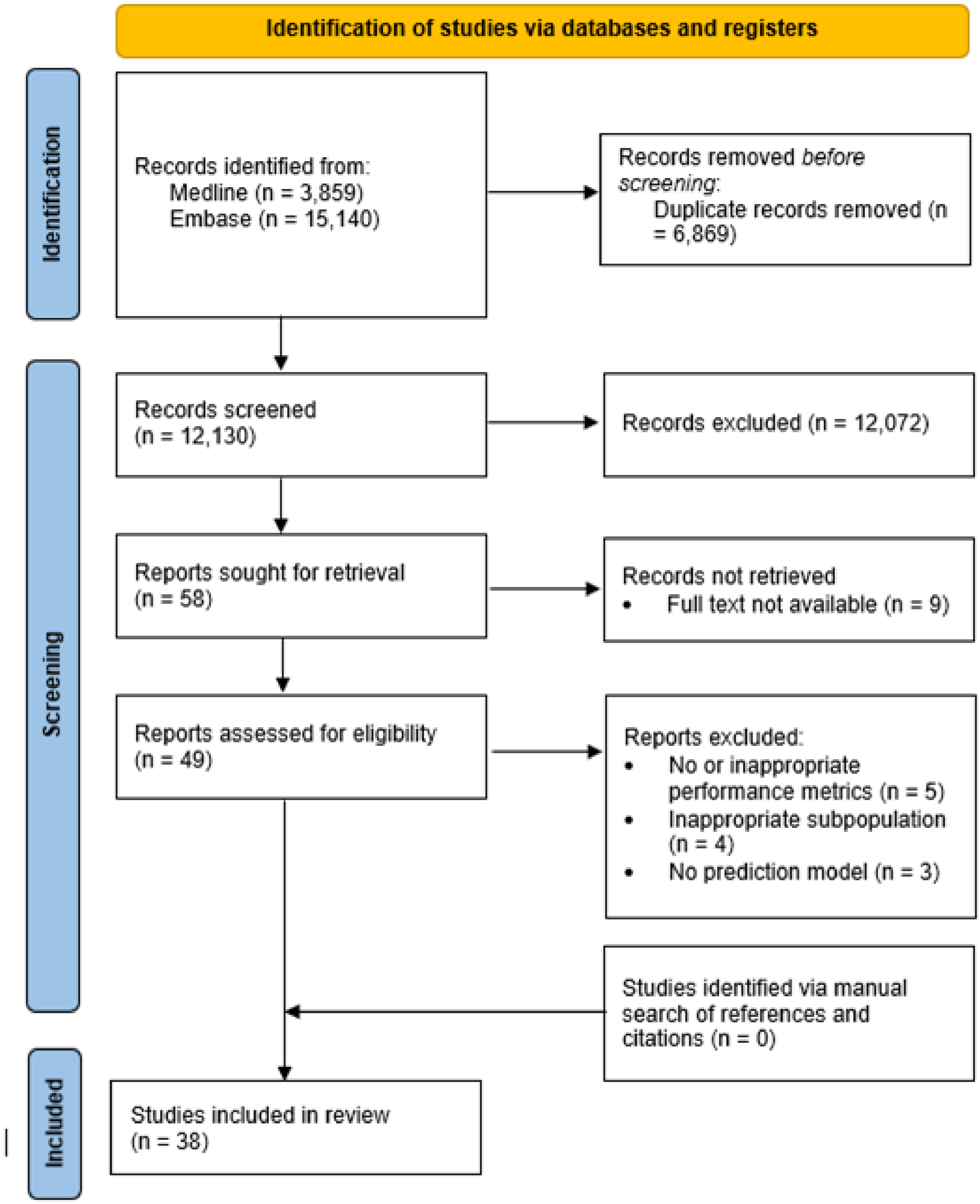

Supplementary Figure 2: Overview of predictor variables used more than once models investigating cardiovascular outcomes

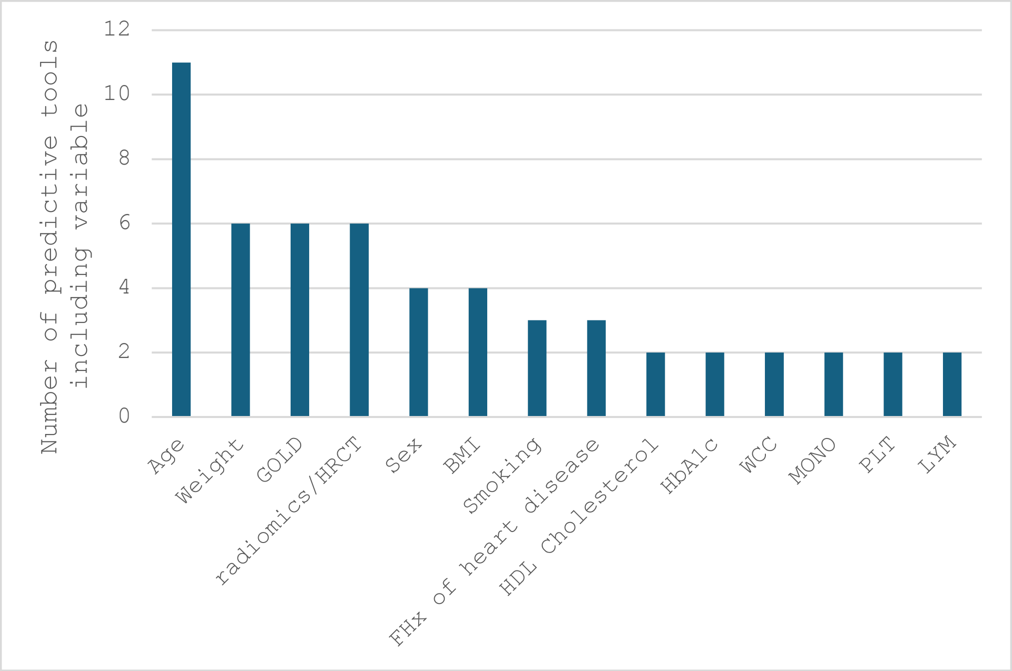

BMI – body mass index; FHx – family history; HbA1c – glycosylated haemoglobin; HDL – high-density lipoprotein; HRCT – high resolution computed tomography; LYM – lymphocytes; MONO – monocytes; PLT – platelets; WCC – white cell count

Supplementary Figure 3: Overview of predictor variables used more than once models investigating exacerbations

BMI – body mass index; CHD – coronary heart disease DM – diabetes mellitus; EOS – serum eosinophils; FEV1 – forced expiratory volume in 1 second; IHD – ischaemic heart disease; LTOT – long term oxygen therapy; mMRC – modified Medical Research Council; WCC – white cell count

Supplementary Figure 4: Overview of predictor variables used more than once models investigating mortality

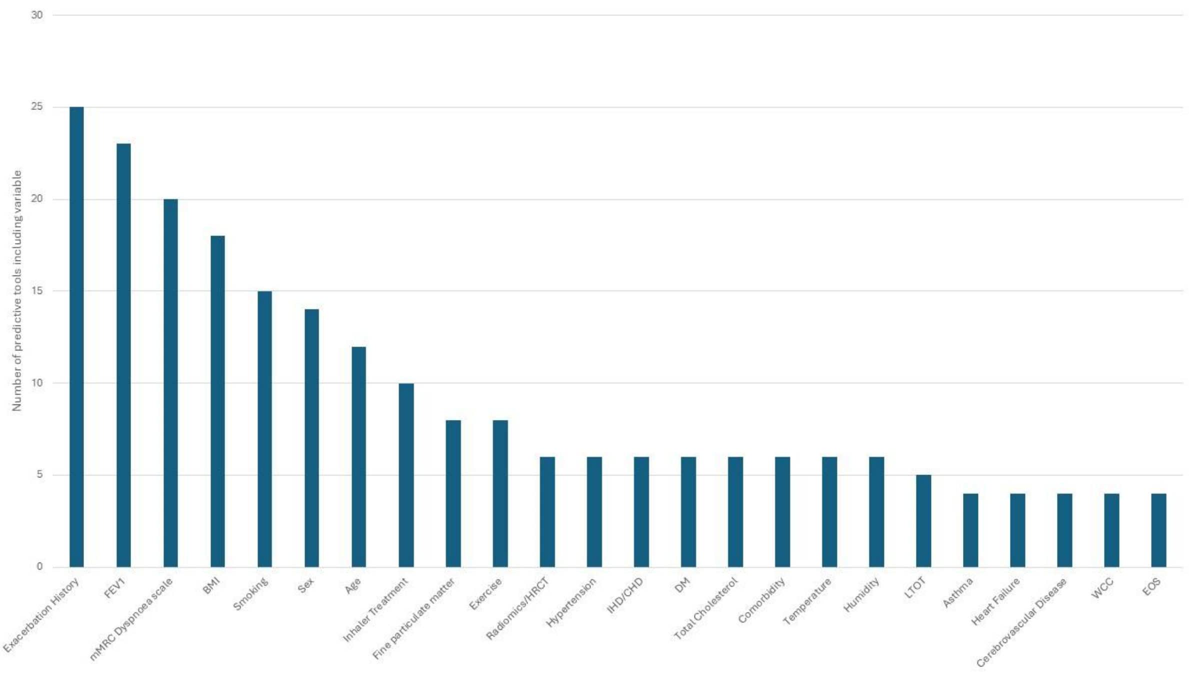

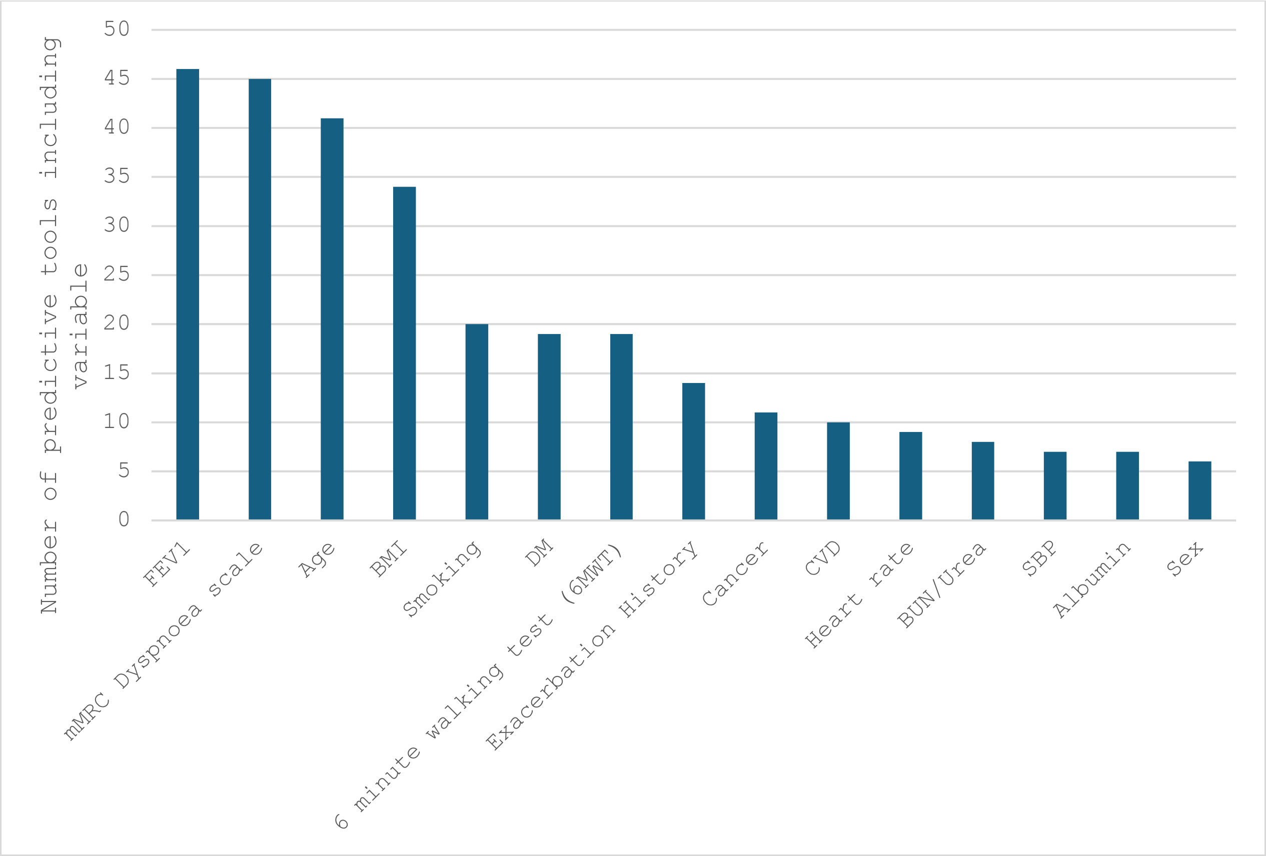

BMI – body mass index; BUN – blood urea nitrogen; CVD – cardiovascular disease; DM – diabetes mellitus; FEV1 – forced expiratory volume in 1 second; mMRC – modified Medical Research Council

Supplementary Figure 5: Risk of Bias assessment for prediction models

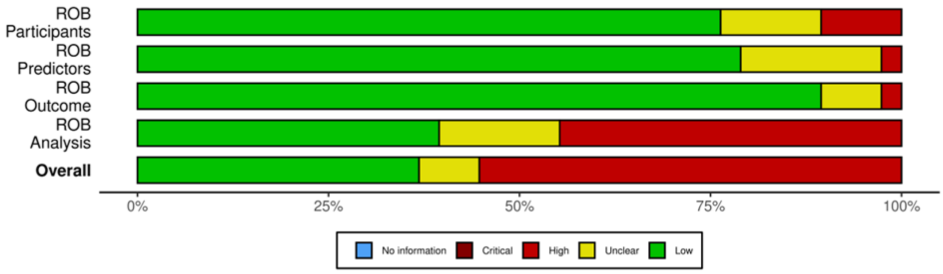

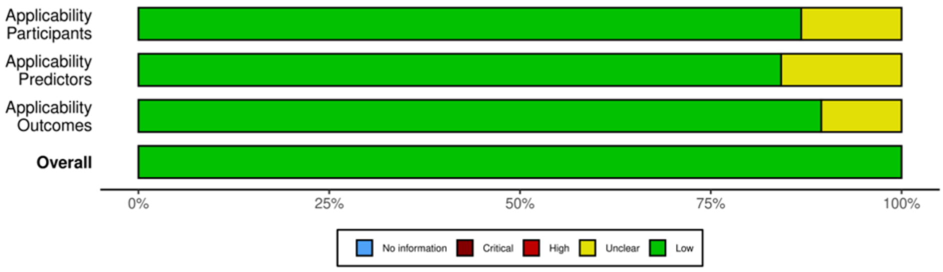

(A) Overall summary of risk of bias for each domain in the systematic review. Green – low risk, yellow – uncertain risk, red – high risk.
(B) Summary of applicability for each domain. Green – low concerns for applicability, amber - unclear concerns for applicability, red – high concerns for applicability

Supplementary Figure 6: Funnel Plot assessing publication bias

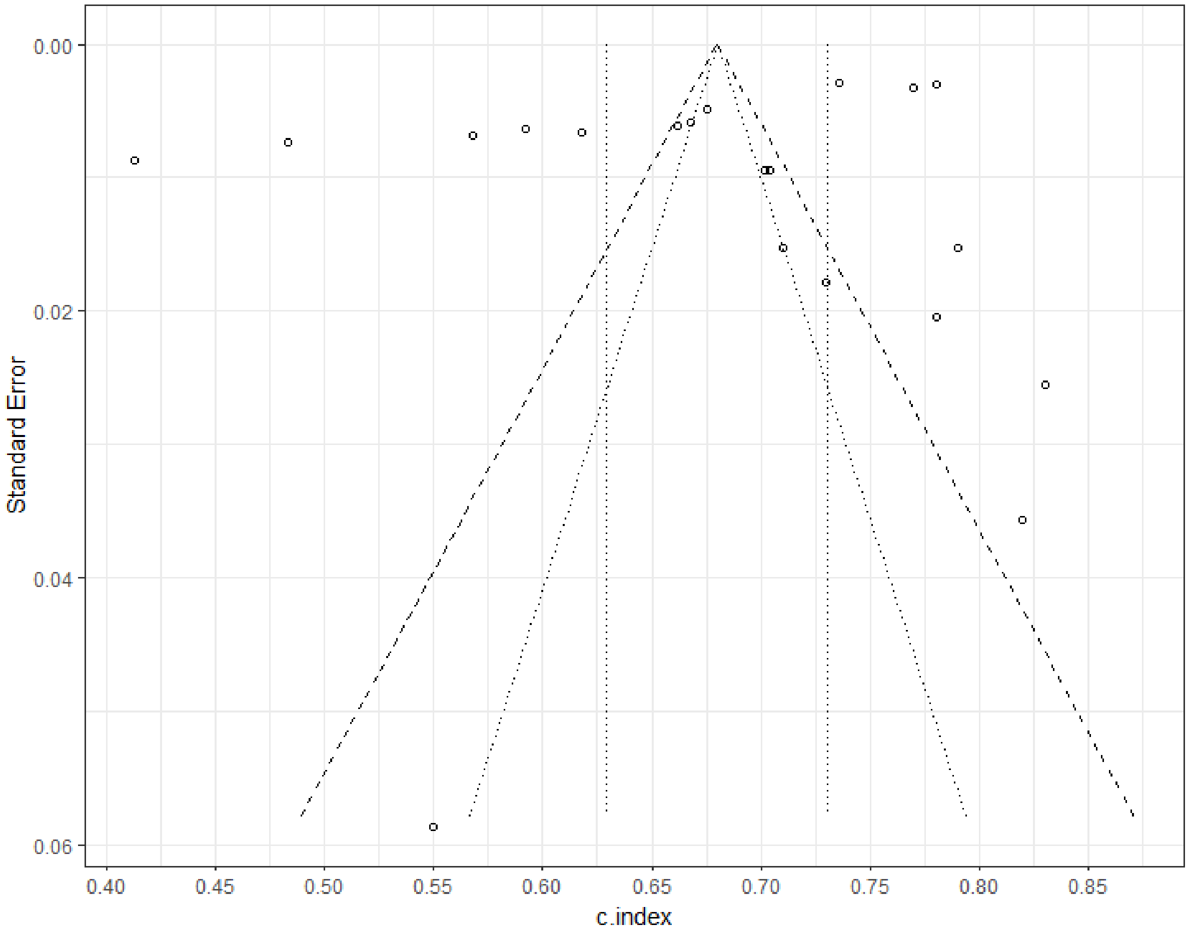

Supplementary Figure 7: Sensitivity analysis for external validation results only for all-cause mortality, excluding studies at high risk of bias.

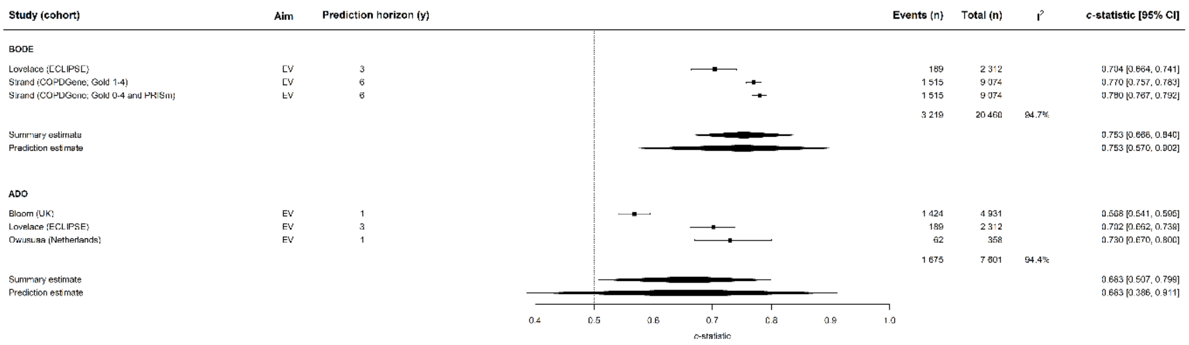

ADO- age, dyspnoea, airflow obstruction; BODE – body mass index, airflow Obstruction, Dyspnea score, and Exercise capacity; BODEx – BODE and exacerbations; EV – external validation;

Supplementary Figure 8: Sensitivity analysis for all-cause mortality restricted to studies with low or unclear risk of bias for participants domain of PROBAST

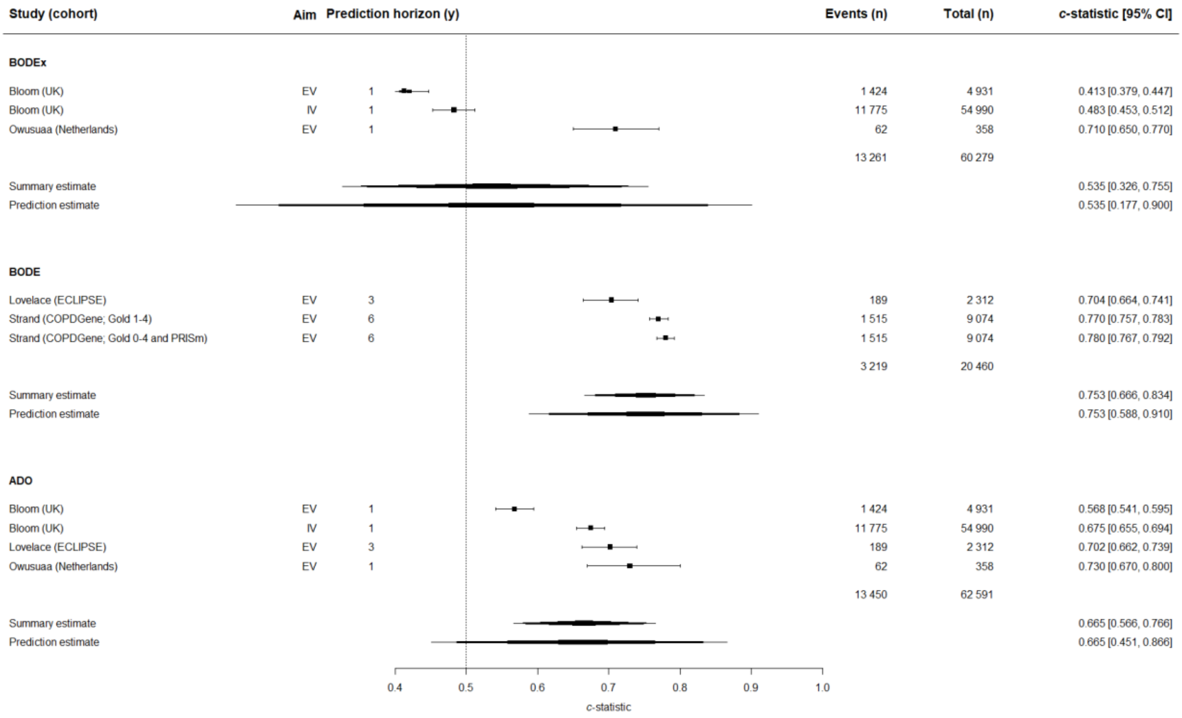

ADO- age, dyspnoea, airflow obstruction; BODE – body mass index, airflow Obstruction, Dyspnea score, and Exercise capacity; BODEx – BODE and exacerbations; EV – external validation; IV – internal validation y - years

References

1. Moons KG, de Groot JA, Bouwmeester W, et al. Critical appraisal and data extraction for systematic reviews of prediction modelling studies: the CHARMS checklist. *PLoS Med* 2014;11(10):e1001744. doi: 10.1371/journal.pmed.1001744 [published Online First: 20141014]
2. Gale CP, Hurst JR, Hawkins NM, et al. Identification and management of cardiopulmonary risk in patients with COPD: a multidisciplinary consensus and modified Delphi study. *Eur J Prev Cardiol* 2025 doi: 10.1093/eurjpc/zwaf119 [published Online First: 20250303]
3. Snell KI, Ensor J, Debray TP, et al. Meta-analysis of prediction model performance across multiple studies: Which scale helps ensure between-study normality for the C-statistic and calibration measures? *Stat Methods Med Res* 2018;27(11):3505-22.
4. metamisc: Meta-Analysis of Diagnosis and Prognosis Research Studies [program], 2020.
5. Debray TP, Damen JA, Riley RD, et al. A framework for meta-analysis of prediction model studies with binary and time-to-event outcomes. *Stat Methods Med Res* 2019;28(9):2768-86.
6. Krishnan S, Tan WC, Farias R, et al. Impaired Spirometry and COPD Increase the Risk of Cardiovascular Disease: A Canadian Cohort Study. *Chest* 2023;164(3):637-49. doi: 10.1016/j.chest.2023.02.045 [published Online First: 20230304]
7. Lin X, Zhou T, Ni J, et al. CT-based whole lung radiomics nomogram: a tool for identifying the risk of cardiovascular disease in patients with chronic obstructive pulmonary disease. *Eur Radiol* 2024;34(8):4852-63. doi: 10.1007/s00330-023-10502-9 [published Online First: 20240112]
8. Huang T, Huang X, Cui X, Dong Q. Predictive nomogram models for atrial fibrillation in COPD patients: A comprehensive analysis of risk factors and prognosis. *Exp Ther Med* 2024;27(4):171. doi: 10.3892/etm.2024.12459 [published Online First: 20240228]
9. Qu S, Zhu J. A Nomogram for Predicting Cardiovascular Diseases in Chronic Obstructive Pulmonary Disease Patients. *J Healthc Eng* 2022;2022:6394290. doi: 10.1155/2022/6394290 [published Online First: 20221018]
10. Shi Y, Zhang J, Huang Y. Prediction of cardiovascular risk in patients with chronic obstructive pulmonary disease: a study of the National Health and Nutrition Examination Survey database. *BMC Cardiovasc Disord* 2021;21(1):417. doi: 10.1186/s12872-021-02225-w [published Online First: 20210901]
11. Abu Hussein NS, Giezendanner S, Urwyler P, et al. Risk Factors for Recurrent Exacerbations in the General-Practitioner-Based Swiss Chronic Obstructive Pulmonary Disease (COPD) Cohort. *J Clin Med* 2023;12(20) doi: 10.3390/jcm12206695 [published Online First: 20231023]
12. Adibi A, Sin DD, Safari A, et al. The Acute COPD Exacerbation Prediction Tool (ACCEPT): a modelling study. *Lancet Respir Med* 2020;8(10):1013-21. doi: 10.1016/S2213-2600(19)30397-2 [published Online First: 20200313]
13. Safari A, Adibi A, Sin DD, et al. ACCEPT 2.0: Recalibrating and externally validating the Acute COPD exacerbation prediction tool (ACCEPT). *EClinicalMedicine* 2022;51:101574. doi: 10.1016/j.eclinm.2022.101574 [published Online First: 20220722]
14. Samp JC, Joo MJ, Schumock GT, et al. Predicting Acute Exacerbations in Chronic Obstructive Pulmonary Disease. *J Manag Care Spec Pharm* 2018;24(3):265-79. doi: 10.18553/jmcp.2018.24.3.265
15. Singla S, Gong M, Riley C, et al. Improving clinical disease subtyping and future events prediction through a chest CT-based deep learning approach. *Med Phys* 2021;48(3):1168-81. doi: 10.1002/mp.14673 [published Online First: 20210127]
16. Wu CT, Li GH, Huang CT, et al. Acute Exacerbation of a Chronic Obstructive Pulmonary Disease Prediction System Using Wearable Device Data, Machine Learning, and Deep Learning: Development and Cohort Study. *JMIR Mhealth Uhealth* 2021;9(5):e22591. doi: 10.2196/22591 [published Online First: 20210506]
17. Bertens LC, Reitsma JB, Moons KG, et al. Development and validation of a model to predict the risk of exacerbations in chronic obstructive pulmonary disease. *Int J Chron Obstruct Pulmon Dis* 2013;8:493-9. doi: 10.2147/COPD.S49609 [published Online First: 20131010]
18. Marin JM, Carrizo SJ, Casanova C, et al. Prediction of risk of COPD exacerbations by the BODE index. *Respir Med* 2009;103(3):373-8. doi: 10.1016/j.rmed.2008.10.004 [published Online First: 20081117]
19. Jones RC, Donaldson GC, Chavannes NH, et al. Derivation and validation of a composite index of severity in chronic obstructive pulmonary disease: the DOSE Index. *Am J Respir Crit Care Med* 2009;180(12):1189-95. doi: 10.1164/rccm.200902-0271OC [published Online First: 20090924]
20. Motegi T, Jones RC, Ishii T, et al. A comparison of three multidimensional indices of COPD severity as predictors of future exacerbations. *Int J Chron Obstruct Pulmon Dis* 2013;8:259-71. doi: 10.2147/COPD.S42769 [published Online First: 20130531]
21. Ko FW, Tam W, Tung AH, et al. A longitudinal study of serial BODE indices in predicting mortality and readmissions for COPD. *Respir Med* 2011;105(2):266-73. doi: 10.1016/j.rmed.2010.06.022 [published Online First: 20100722]
22. Moberg M, Vestbo J, Martinez G, et al. Validation of the i-BODE index as a predictor of hospitalization and mortality in patients with COPD participating in pulmonary rehabilitation. *COPD* 2014;11(4):381-7. doi: 10.3109/15412555.2013.836171 [published Online First: 20131010]
23. Li J, Liang L, Samuel Cai Y, et al. Tracking COPD exacerbation patterns and forecasting readmission risks utilizing electronic medical records. *Int J Med Inform* 2024;189:105505. doi: 10.1016/j.ijmedinf.2024.105505 [published Online First: 20240531]
24. Wang S, Li W, Zeng N, et al. Acute exacerbation prediction of COPD based on Auto-metric graph neural network with inspiratory and expiratory chest CT images. *Heliyon* 2024;10(7):e28724. doi: 10.1016/j.heliyon.2024.e28724 [published Online First: 20240329]
25. Chen SY, Huang CK, Wu CL, et al. Prognostic value of the post-exercise heart rate recovery and BHDE-index in chronic obstructive pulmonary disease. *BMC Pulm Med* 2023;23(1):263. doi: 10.1186/s12890-023-02557-7 [published Online First: 20230717]
26. Singh D, Hurst JR, Martinez FJ, et al. Predictive modeling of COPD exacerbation rates using baseline risk factors. *Ther Adv Respir Dis* 2022;16:17534666221107314. doi: 10.1177/17534666221107314
27. Huang ZY, Lin S, Long LL, et al. Predicting the morbidity of chronic obstructive pulmonary disease based on multiple locally weighted linear regression model with K-means clustering. *Int J Med Inform* 2020;139:104141. doi: 10.1016/j.ijmedinf.2020.104141 [published Online First: 20200414]

28. Crutsen MRC, Keene SJ, Nakken D, et al. Physical, Psychological, and Social Factors Associated with Exacerbation-Related Hospitalization in Patients with COPD. *J Clin Med* 2020;9(3) doi: 10.3390/jcm9030636 [published Online First: 20200227]
29. Chen X, Wang Q, Hu Y, et al. A Nomogram for Predicting Severe Exacerbations in Stable COPD Patients. *Int J Chron Obstruct Pulmon Dis* 2020;15:379-88. doi: 10.2147/COPD.S234241 [published Online First: 20200218]
30. Yii ACA, Loh CH, Tiew PY, et al. A clinical prediction model for hospitalized COPD exacerbations based on "treatable traits". *Int J Chron Obstruct Pulmon Dis* 2019;14:719-28. doi: 10.2147/COPD.S194922 [published Online First: 20190327]
31. Lovelace TC, Ryu MH, Jia M, et al. Development and validation of a mortality risk prediction model for chronic obstructive pulmonary disease: a cross-sectional study using probabilistic graphical modelling. *EClinicalMedicine* 2024;75:102786. doi: 10.1016/j.eclinm.2024.102786 [published Online First: 20240822]
32. Shah SA, Nwaru BI, Sheikh A, et al. Development and validation of a multivariable mortality risk prediction model for COPD in primary care. *NPJ Prim Care Respir Med* 2022;32(1):21. doi: 10.1038/s41533-022-00280-0 [published Online First: 20220531]
33. Huang D, He D, Gong L, et al. A prediction model for hospital mortality in patients with severe community-acquired pneumonia and chronic obstructive pulmonary disease. *Respir Res* 2022;23(1):250. doi: 10.1186/s12931-022-02181-9 [published Online First: 20220918]
34. Pellicori P, McConnachie A, Carlin C, et al. Predicting mortality after hospitalisation for COPD using electronic health records. *Pharmacol Res* 2022;179:106199. doi: 10.1016/j.phrs.2022.106199 [published Online First: 20220404]
35. Owusuaa C, van der Leest C, Helfrich G, et al. The development of the ADO-SQ model to predict 1-year mortality in patients with COPD. *Palliat Med* 2022;36(5):821-29. doi: 10.1177/02692163221080662 [published Online First: 20220324]
36. Bloom CI, Ricciardi F, Smeeth L, et al. Predicting COPD 1-year mortality using prognostic predictors routinely measured in primary care. *BMC Med* 2019;17(1):73. doi: 10.1186/s12916-019-1310-0 [published Online First: 20190405]
37. Tsimogianni AM, Papiris SA, Stathopoulos GT, et al. Predictors of outcome after exacerbation of chronic obstructive pulmonary disease. *J Gen Intern Med* 2009;24(9):1043-8. doi: 10.1007/s11606-009-1061-2 [published Online First: 20090714]
38. Sun W, Li Y, Tan S. Development and Validation of an In-Hospital Mortality Prediction Model for Patients with Acute Exacerbation of Chronic Obstructive Pulmonary Disease. *Int J Chron Obstruct Pulmon Dis* 2024;19:1303-14. doi: 10.2147/COPD.S461269 [published Online First: 20240612]
39. Horne BD, Hegewald MJ, Crim C, et al. The Summit Score Stratifies Mortality and Morbidity in Chronic Obstructive Pulmonary Disease. *Int J Chron Obstruct Pulmon Dis* 2020;15:1741-50. doi: 10.2147/COPD.S254437 [published Online First: 20200720]
40. Liao KM, Liu CF, Chen CJ, Shen YT. Machine Learning Approaches for Predicting Acute Respiratory Failure, Ventilator Dependence, and Mortality in Chronic Obstructive Pulmonary Disease. *Diagnostics (Basel)* 2021;11(12) doi: 10.3390/diagnostics11122396 [published Online First: 20211220]
41. Ryynanen OP, Soini EJ, Lindqvist A, et al. Bayesian predictors of very poor health related quality of life and mortality in patients with COPD. *BMC Med Inform Decis Mak* 2013;13:34. doi: 10.1186/1472-6947-13-34 [published Online First: 20130307]
42. Dong F, Ren X, Huang K, et al. Development and Validation of Risk Prediction Model for In-hospital Mortality Among Patients Hospitalized With Acute Exacerbation Chronic Obstructive Pulmonary Disease Between 2015 and 2019. *Front Med (Lausanne)* 2021;8:630870. doi: 10.3389/fmed.2021.630870 [published Online First: 20210406]
43. Strand M, Austin E, Moll M, et al. A Risk Prediction Model for Mortality Among Smokers in the COPDGene(R) Study. *Chronic Obstr Pulm Dis* 2020;7(4):346-61. doi: 10.15326/jcopdf.7.4.2020.0146
44. Lovelace TC, Ryu MH, Jia M, et al. Disentangling Predictors of COPD Mortality with Probabilistic Graphical Models. *medRxiv* 2024 doi: 10.1101/2024.01.31.24301705 [published Online First: 20240201]
